# Quantifying the roles of host movement and vector dispersal in the transmission of vector-borne diseases of livestock

**DOI:** 10.1371/journal.pcbi.1005470

**Published:** 2017-04-03

**Authors:** Tom Sumner, Richard J. Orton, Darren M. Green, Rowland R. Kao, Simon Gubbins

**Affiliations:** 1 The Pirbright Institute, Pirbright, Surrey, United Kingdom; 2 Boyd Orr Centre for Population and Ecosystem Health, Institute of Biodiversity, Animal Health and Comparative Medicine, College of Medical, Veterinary and Life Sciences, University of Glasgow, Glasgow, United Kingdom; 3 Institute of Aquaculture, University of Stirling, Stirling, Stirlingshire, United Kingdom; University of California, Los Angeles, UNITED STATES

## Abstract

The role of host movement in the spread of vector-borne diseases of livestock has been little studied. Here we develop a mathematical framework that allows us to disentangle and quantify the roles of vector dispersal and livestock movement in transmission between farms. We apply this framework to outbreaks of bluetongue virus (BTV) and Schmallenberg virus (SBV) in Great Britain, both of which are spread by *Culicoides* biting midges and have recently emerged in northern Europe. For BTV we estimate parameters by fitting the model to outbreak data using approximate Bayesian computation, while for SBV we use previously derived estimates. We find that around 90% of transmission of BTV between farms is a result of vector dispersal, while for SBV this proportion is 98%. This difference is a consequence of higher vector competence and shorter duration of viraemia for SBV compared with BTV. For both viruses we estimate that the mean number of secondary infections per infected farm is greater than one for vector dispersal, but below one for livestock movements. Although livestock movements account for a small proportion of transmission and cannot sustain an outbreak on their own, they play an important role in establishing new foci of infection. However, the impact of restricting livestock movements on the spread of both viruses depends critically on assumptions made about the distances over which vector dispersal occurs. If vector dispersal occurs primarily at a local scale (99% of transmission occurs <25 km), movement restrictions are predicted to be effective at reducing spread, but if dispersal occurs frequently over longer distances (99% of transmission occurs <50 km) they are not.

## Author summary

Diseases which are transmitted by the bites of insects can be spread to new locations through the movement of both infected insects and infected hosts. The importance of these routes has implications for disease control, because we can often restrict host movement, and so potentially reduce spread, but cannot easily restrict insect movements. Despite this, the importance of host movements has been little studied. Here we develop a mathematical model which allows us to disentangle and quantify transmission by insect dispersal and by host movement. We apply the model to two diseases of cattle and sheep transmitted by biting midges that have emerged in northern Europe in the past decade, bluetongue virus (BTV) and Schmallenberg virus (SBV). For both viruses, we show insect movements account for a majority of spread between farms. Although they cannot sustain an epidemic on their own, animal movements play an important role in introducing disease to new areas.

## Introduction

The role of host movements in the transmission of vector-borne diseases has largely been ignored [1,2]. Recently, however, several studies have quantified the importance of human movements for the spread of vector-borne diseases. Mobile phone data were used to infer mobility patterns and, hence, the impact of large-scale movement patterns on the transmission of malaria [3] and dengue [4]. Alternatively, detailed social surveys were used to investigate the importance of house-to-house movements on dengue virus transmission [5,6].

Both approaches exemplify a central difficulty in studying the role of human movement in the transmission of vector-borne diseases: they must rely on detailed small-scale studies or proxy measures, because human movements are seldom recorded in detail [2]. By contrast, livestock movements, and those of cattle in particular, are well described in many countries. The role of livestock movements in the spread of infectious diseases has been widely studied for directly-transmitted infections, such as bovine tuberculosis [7–9] or foot-and-mouth disease [10–12]. However, their role in the spread of vector-borne diseases has not been explored in any great detail.

In this study we disentangle and quantify the relative importance of livestock movements and vector dispersal in the spread of two viral infections of cattle and sheep, both of which are transmitted by *Culicoides* biting midges and have recently emerged in northern Europe. In 2006 bluetongue virus (BTV) serotype 8 (BTV-8) appeared near Maastricht in the Netherlands and, subsequently, spread to much of northern Europe [13,14]. Schmallenberg virus (SBV), a novel orthobunyavirus, was first detected in Germany and the Netherlands in the summer of 2011 [15] and by spring of 2013 had been reported across much of Europe [16].

We develop a model describing the transmission of BTV and SBV within and between farms, which uses separate descriptions for transmission via dispersal of infected vectors and via movement of infected livestock. First, we apply the model to demographic and epidemiological data from the BTV-8 epidemic in Great Britain (GB) during 2007 in a Bayesian framework. This approach allows us to link (unobserved) infection with reported clinical disease. It also allows us to update previous estimates for epidemiological parameters related to the transmission of BTV within and between farms (cf. [17,18]). Next, the model is applied to SBV using previously derived estimates relating to transmission within a farm [19]. Finally, the practical implications of the results are explored by assessing the impact of controlling livestock movements on the spread of the two viruses.

## Methods

### Data sources

#### Demographic data

The location and number of cattle and sheep on each farm were obtained from June agricultural survey data for 2006. Animal movement data for 2006 and 2007 were extracted from the Cattle Tracing System (CTS) for cattle, from the Animal Movements Licensing System (AMLS) for sheep in England and Wales and from the Scottish Animal Movements System (SAMS) for sheep in Scotland. The movement data for 2006 represent a normal year for animal movements (there were no major disease outbreaks), while the movement data for 2007 incorporate the movement restrictions imposed during the BTV (and foot-and-mouth disease) outbreak.

#### Epidemiological data

Summary outbreak measures for BTV in GB during 2007 were extracted from the Defra epidemiology report [20] and are provided in S1 Data. Six measures were used:

1. number of newly confirmed clinical farms each week between 4 August 2007 (the most likely date of the initial incursion [21]) and 19 December 2007 (there were no confirmed clinical cases after this date);
2. cumulative number of confirmed clinical farms in each county (up to 19 December 2007);
3. number of infected farms identified by pre-movement testing during the so-called “vector-free” period (VFP) declared by Defra between 20 December 2007 and 15 March 2008;
4. number of infected farms identified by targeted surveillance around the first infected premises (IP) near Ipswich, where all cattle farms within 3 km and cattle farms with more than 50 animals between 3 km and 10 km of the IP were tested;
5. number of infected farms identified by targeted surveillance around the first IP near Lowestoft, where all cattle farms within 5 km and cattle farms with more than 50 animals between 5 km and 10 km of the IP were tested; and
6. mean within-farm prevalence (11%) for 39 cattle herds in East Anglia sampled in January 2008.

#### Initial conditions for 2007

To seed the model nine farms were assumed to be independently infected. Seven were the earliest reported cases in their county and were assumed to be a result of windborne incursions of infected vectors from continental Europe. Of these, two farms in Suffolk were infected on 4 August 2007, three farms in Kent and one farm in East Sussex were infected on 12 September 2007 and one farm in Essex was infected on 13 September. The timing of these incursions is based on a retrospective analysis of suitable meteorological conditions for the introduction of BTV-infected midges [21]. The remaining two affected farms were both in Cambridgeshire and had no identifiable link with the main outbreak areas and these were assumed to be infected 10 days prior to their report date.

#### Climate data

Temperature data were obtained from the UK Climate Projections (UKCP09) gridded observation data-sets for 2006, 2007 and 2011. These cover the UK at 5 km × 5 km resolution, with farms using the temperature data for the grid square in which they are located.

### Within-farm transmission of bluetongue virus

The dynamics of BTV within a farm are described using a stochastic compartmental model that includes two ruminant host species (cattle and sheep) and the *Culicoides* vector [18].

The cattle and sheep populations are assumed to be constant (*H*_*i*_), except for disease-associated mortality, and are subdivided into the number of susceptible (i.e. uninfected), infected and recovered animals, denoted by *X*^(*i*)^, *Y*^(*i*)^ and *Z*^(*i*)^, respectively, where the superscript *i* indicates cattle (*C*) or sheep (*S*). To allow for a more general gamma distribution for the duration of viraemia, the infected host population, *Y*^(*i*)^, is subdivided into a number of stages, with newly infected hosts entering the first stage and then passing through each successive stage. If the time spent in each stage follows an exponential distribution with mean 1/*n*_*i*_*r*_*i*_, the total length of time spent in the *n*_*i*_ stages follows a gamma distribution, with mean 1/*r*_*i*_ and variance 1/*n*_*i*_*r*_*i*_^2^ [22].

The vector population (*N*) is subdivided into the number of adult female midges that are susceptible (i.e. uninfected), latent (i.e. infected, but not infectious) and infectious, denoted by *S*, *L* and *I*, respectively. To allow for a more general gamma distribution for the extrinsic incubation (i.e. latent) period (EIP) [23], the latent class is subdivided into a number of stages in a similar approach to that described above for the duration of host viraemia. Vector mortality occurs at the same rate in all classes and is balanced by the recruitment of susceptible vectors, so that the total vector population (*N*) remains constant.

The force of infection for host species *i*, *λ*_*i*_, is given by,

$$\lambda_{i}(t)=ba\phi_{i}m_{i}\theta (t)\frac{I(t)}{N},$$ (1)

where *b* is the probability of transmission from an infected vector to a host, *a* is the reciprocal of the time interval between blood meals for the vector (assumed to be equal to the biting rate), *m*_*i*_ (= *N*/*H*_*i*_) is the vector-to-host ratio and *I*/*N* is the proportion of bites which are from infectious vectors. The proportion of bites on cattle and sheep is given by

$$\phi_{C}=\frac{H_{C}}{H_{C}+\sigma H_{S}},\;\phi_{S}=1-\phi_{C},$$ (2)

respectively, where *σ* is the vector preference for sheep relative to cattle. The seasonal vector activity [24] on day *t* is given by

$$\theta (t)\propto \text{exp}\left(b_{11}\text{sin}\left(\frac{2\pi t}{365}\right)+b_{21}\text{cos}\left(\frac{2\pi t}{365}\right)+b_{12}\text{sin}\left(\frac{4\pi t}{365}\right)+b_{22}\text{cos}\left(\frac{4\pi t}{365}\right)\right),$$ (3)

normalised so the maximum value is one. The force of infection for vectors, *λ*_*V*_, is

$$\lambda_{V}(t)=\beta a\theta (t)\left(\phi_{C}\frac{Y^{\left(C\right)}(t)}{H_{C}}+\phi_{S}\frac{Y^{\left(S\right)}(t)}{H_{S}}\right),$$ (4)

where *β* is the probability of transmission from an infected host to a vector and *Y*^(*C*)^ and *Y*^(*S*)^ are the total number of infected cattle and sheep, respectively.

Infection on a farm was related to reported clinical disease by assuming there was a daily probability of a farm with infected cattle or sheep reporting clinical disease, *ζ*_*C*_ and ζ_*S*_, respectively, where 0≤*ζ*_*C*_,ζ_*S*_≤1.

Parameters in the model are summarised in S1 Table. The reciprocal of the time interval between blood meals (*a*), the vector mortality rate (*μ*) and the reciprocal of the mean EIP (*ν*) were assumed to vary with the local temperature (see S1 Table for details).

Population sizes in the model take integer values, while transitions between compartments are stochastic processes (S2 Table). The number of transitions of each type during a small time interval *δt* was drawn from a binomial distribution with population size *n* and transition probability *q* (the appropriate *per capita* rate multiplied by *δt*) (S2 Table). However, binomial random variables are computationally expensive to simulate and an approximating distribution was used wherever possible. If: (i) *nq*(1-*q*)>25; (ii) *nq*(1-*q*)>5 and 0.1<*q*<0.9; or (iii) min(*nq*,*n*(1-*q*))>10, an approximating normal variate with mean *nq* and variance *nq*(1-*q*) was used, while if *q*<0.1 and *nq*<10, an approximating Poisson variate with mean *nq* was used [25].

### Transmission of bluetongue virus between farms

To describe the spread of BTV between farms, a stochastic, spatially-explicit model with a daily time-step was used. Transmission between farms was assumed to occur via two routes: movement of infected animals or dispersal of infected vectors.

#### Movement of infected livestock

Movement of infected livestock was modelled by the following sequence of steps. For each farm with infected cattle or sheep:

1. determine the number of batches of animals moved off the farm that day, which depends on the number of animals on the farm and on the month;
2. for each batch determine the batch size (i.e. number of animals moved) and then determine the number of infected animals in the batch (sampling without replacement);
3. if there is at least one infected animal in the batch, determine where it is moved to:
  - select the county to which the batch is moved based on the relative frequency of movements from the county in which the farm is located to all counties (including that in which the farm is located);
  - select a herd or flock at random from the county and test if it buys-in animals that day (repeating as necessary until a farm does buy-in animals), where the probability depends on the number of animals on the recipient farm and on the month;
  - if the herd or flock buying-in animals is uninfected, it acquires infection (i.e. the number of infected animals in the batch).

The distributions and parameters required for each step are described in detail in S1 Text. Parameters were estimated using data on recorded cattle and sheep movements for GB (S3 and S4 Tables).

#### Dispersal of infected vectors

Long-distance dispersal of *Culicoides* biting midges over water has been linked to incursions of *Culicoides*-borne viruses to islands hundreds of kilometres from the nearest source of virus, where introduction via animal movements had been discounted [26–29]. By contrast, dispersal over land, as inferred from outbreak data, typically follows a stepping-stone pattern, with limited evidence of single, long-distance dispersal events [30–32]. This pattern of spread is consistent with *Culicoides* dispersal over land being a mixture of appetitive upwind flight towards host and habitat cues and short-range, wind-assisted flight [31,33].

Accordingly, we first model dispersal of infected vectors between farms as a diffusion process (cf. [34]), adapted to allow for seasonal variation in vector activity and to incorporate the probability that a dispersing midge will survive for long enough to reach an at-risk farm. In this case, the force of infection that farm *j* exerts on farm *k* on day *t* was given by

$$\begin{array}{l} \lambda (x_{jk},t)=\gamma \sum_{t^{\prime }=\tau_{j}}^{t}\{\theta (t^{\prime })I(t^{\prime })\times \text{exp}\left(-\sum_{t^{\prime \prime }=t^{\prime }}^{t}\mu (T(t^{\prime \prime }))\right)\times \\ \;\frac{1}{4\pi D(t-t^{\prime }+1)}\text{exp}\left(-\frac{x_{jk}^{2}}{4D(t-t^{\prime }+1)}\right)\}, \end{array}$$ (5)

where *γ* is the transmission parameter, *τ*_*j*_ is the day on which infectious vectors were first present on farm *j*, *θ*(*t*) is seasonal vector activity (given by (Eq 3)), *I*(*t*) is the number of infectious vectors on the farm, *μ*(*T*(*t*)) is the (temperature-dependent) vector mortality rate (see S1 Table), *D* is the diffusion coefficient and *x*_*jk*_ is the distance between the farms.

To explore the influence of assumptions about dispersal distances on model predictions for transmission via dispersal of infected vectors, we also used a suite of dispersal kernels to describe transmission by this route. In this case, the force of infection was given by

$$\lambda (x_{jk},t)=\gamma \theta (t)I(t)K(x_{jk}),$$ (6)

where *γ* is the transmission parameter, *θ*(*t*) is seasonal vector activity (given by (Eq 3)), *I*(*t*) is the proportion of infectious vectors and *K*(*x*) is the dispersal kernel. Four forms for the kernel were considered: exponential, Gaussian, fat-tailed and stepped (Table 1). The first three have been used previously to describe the spread of BTV between farms [18,35], while the stepped kernel allows for more intense short-range (i.e. local) dispersal coupled with less frequent longer-range dispersal.

**Table 1:** Summary of models for transmission of bluetongue virus between farms via dispersal of infectious vectors.

model	function	parameters	priors
diffusion	see Eq (5)	diffusion parameter (*D*)	Uniform(0,5) [34]
exponential kernel	*K*(*x*) = exp(−*αx*)	kernel parameter (*α*)	Exponential(0.06) [18]
Gaussian kernel	*K*(*x*) = exp(−*αx*^2^)	kernel parameter (*α*)	Exponential(0.0012) [18]
fat-tailed kernel	$K(x)=\frac{1}{1+{\left(\frac{x}{d_{0}}\right)}^{\alpha }}$	kernel power (*α*) distance scaling (*d*_0_)	Uniform(1,5) Exponential(10) [35]
stepped kernel	$K(x)=\{\begin{array}{c} \;1\;x\leq d_{0}\\ {\left(\frac{x}{d_{0}}\right)}^{-\alpha }\;x>d_{0} \end{array}$	kernel power (*α*) distance parameter (*d*_0_)	Uniform(1,5) Exponential(10) [35]

For both the diffusion and kernel models, the probability that farm *j* infects farm *k* on day *t* via dispersal of infected vectors is

$$p_{jk}(t)=1-\text{exp}\left(-\lambda (x_{jk},t)\right),$$ (7)

where *λ*(*x*_*jk*_,*t*) is the force of infection given by Eqs (5) or (6).

### Approximate Bayesian computation

A total of 22 or 23 parameters were estimated by fitting the BTV model to the summary outbreak data for Great Britain in 2007: two or three related to vector dispersal (depending on the model used; Table 1); two related to under-ascertainment of infected farms (*ζ*_*C*_ and *ζ*_*S*_); and 18 related to within-farm transmission (see S1 Table).

#### ABC-SMC sampling

Parameters were estimated using approximate Bayesian computation (ABC) sequential Monte Carlo (SMC) sampling [36,37]. This replaces the full likelihood for a model (which would be too complex to calculate in the case of the BTV model) with an approximation based on matching model simulations to observed data using a set of goodness-of-fit metrics (defined below for the BTV model). The ABC-SMC algorithm (described in more detail in S2 Text) begins by generating a number of particles, or parameter sets, from the joint prior distribution (in the case of the BTV model, we used 10,000 particles and the priors are defined below). For each particle, a model simulation is run and a binary Monte Carlo estimate of the probability of matching is produced (i.e. whether or not the goodness-of-fit metrics for the model simulation lie within a specified tolerance). These estimates are combined with the prior probabilities to produce a set of weights for the particles. The algorithm then proceeds through a series of rounds in which the particles are resampled based on the weights from the previous round and perturbed according to a pre-defined perturbation kernel. A new set of weights is calculated in a similar manner to before. The tolerance controlling the matching is reduced at each step until the model simulations generate an acceptable agreement with the observed epidemiological measures.

#### Goodness-of-fit metric

The six summary outbreak measures described in the Data sources section were used to judge model fit. More specifically, chi-squared goodness-of-fit metrics for each measure (cf. [37]) were combined to produce a single metric, *M*, given by

$$\begin{array}{lll} M & = & \sum_{w}\frac{{\left(C_{w}^{\left(sim\right)}-C_{w}^{\left(obs\right)}\right)}^{2}}{C_{TOT}^{\left(obs\right)}}+\sum_{r}\frac{{\left(C_{r}^{\left(sim\right)}-C_{r}^{\left(obs\right)}\right)}^{2}}{C_{TOT}^{\left(obs\right)}}+\\ & & \sum_{j}\frac{{\left(I_{j}^{\left(sim\right)}-I_{j}^{\left(obs\right)}\right)}^{2}}{I_{j}^{\left(obs\right)}}+\frac{{\left(p_{sim}-p_{obs}\right)}^{2}}{p_{obs}}, \end{array}$$ (8)

where *C*_*w*_ is the number of reported clinical cases each week, *C*_*r*_ is the cumulative number of reported cases in each county, *C*_*TOT*_ is the total number of reported cases, *I*_*j*_ is the number of infected holdings detected by pre-movement testing (*j* = PMT) or by targeted surveillance around IPs near Ipswich (*j* = TS1) or Lowestoft (*j* = TS2), *p* is the mean within-farm prevalence for cattle herds and *sim* and *obs* indicate simulated and observed measures, respectively.

When simulating the model the number of infected farms reporting clinical disease each day was aggregated to calculate the number of infected farms reporting clinical disease each week and the cumulative number of infected farms reporting clinical disease in each county. The number of infected farms detected by pre-movement testing during the VFP was calculated by simulating movements during the VFP for each infected farm which did not report clinical disease. If at least one infected animal was moved, the farm was assumed to be detected. The number of infected farms detected by targeted surveillance around the first IPs near Ipswich or Lowestoft was computed by testing whether cattle farms of the appropriate size and within the appropriate distance of the initially infected farms were infected before 19 October 2007. Finally, the mean within-farm prevalence was computed for all infected cattle herds.

#### Prior distributions

A mildly-informative Exponential(1) prior was used for the vector transmission rate (γ), while priors with means derived from previous estimates were used for each of the vector dispersal models (see Table 1). Uniform(0,0.2) priors were used for the two parameters related to under-ascertainment (*ζ*_*C*_ and *ζ*_*S*_). Finally, informative priors for the 18 parameters related to within-farm transmission were constructed based on the published literature (see S1 Table). All priors were assumed to be independent of one another.

Parameters were common to all farms, except for the vector-to-host ratio which was sampled independently from a gamma distribution (with mean and shape parameter estimated as part of model fitting) for each farm.

### Applying the model to SBV

When applying the model framework to SBV, estimates for parameters related to the transmission of SBV within a farm were derived from an earlier study in which the within-farm component of the model was fitted to sero-prevalence data for Belgium and the Netherlands [19]. Parameters for transmission between farms were assumed to be the same as for BTV.

Unlike for BTV, the timing of incursions for SBV in GB has not been investigated in any great detail. Back-calculation from the dates of reported cases of malformed calves and lambs indicates that a plausible date for an incursion is 28 June 2011 [38], though it is not possible to rule out earlier dates or, indeed, multiple dates of incursion. For simplicity, each incursion was initialised on the 28 June by selecting a single farm at random from a county on the south-east coast of England (Suffolk, Essex, Kent, East Sussex, West Sussex, Hampshire and Isle of Wight), which were the earliest affected regions. The aim here is to compare the importance of transmission routes for SBV with those for BTV, rather than to reconstruct the SBV epidemic in GB. However, the sensitivity of the results for SBV to the timing of incursion was assessed by simulating incursions on five other incursion dates throughout the year (1 May, 1 June, 1 July, 1 August and 1 September).

### Impact of movement restrictions on spread

To explore the impact of movement restrictions on the spread of BTV and SBV we assumed they were applied in a circular zone around known IPs. For BTV IPs were detected on the basis of reported clinical disease, while for SBV they were assumed to be detected when the first newly infected cattle or sheep occurred on the farm (adult animals show no or very mild clinical signs of disease). Farms within a specified radius of an IP became part of the movement restriction zone (MRZ) and were allowed to move animals to farms within the MRZ, but not to any farms outside the MRZ. For each radius, one hundred replicates of the model were run for five incursion dates (1 May, 1 June, 1 July, 1 August and 1 September) until 31 December. Each incursion was initialised by selecting a single farm at random from a county on the south-east coast of England (Suffolk, Essex, Kent, East Sussex or West Sussex). The model for transmission between farms via animal movements was parameterised using movement data for 2006 (i.e. a year in which there were no major outbreaks of disease in cattle or sheep). We explored the sensitivity of the model predictions for the effectiveness of movement controls to temperature by using data for two years: 2006 (a warmer year) and 2007 (a cooler year).

## Results

### Observed and predicted dynamics of BTV-8 in GB

**Fig 1:**
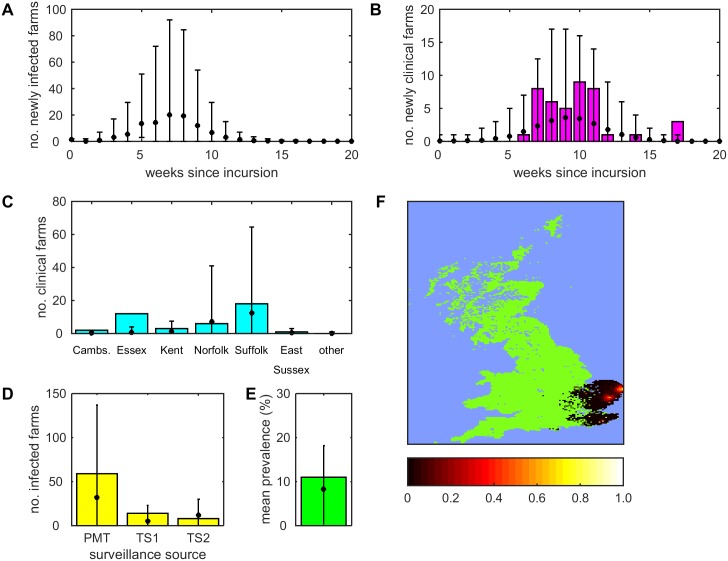
Observed and predicted spread of bluetongue virus serotype 8 in Great Britain in 2007 for the model in which vector dispersal is described by a diffusion process. (A, B) Number of (A) newly infected farms and (B) farms with newly confirmed clinical cases each week. (C) Cumulative number of farms with confirmed clinical cases in each county. The six named counties are those for which there were confirmed clinical cases. (D) Number of infected farms detected by pre-movement testing (PMT) or targeted surveillance around the first two affected farms in Suffolk (TS1 and TS2). (E) Within-farm prevalence for cattle herds. Each plot shows the observed values (bars) and mean (circles) and 95% prediction intervals for the simulations. (F) Predicted spatial spread of BTV-8 in GB. The map shows the cumulative probability of infection (see scale bar) expressed as the proportion of simulated outbreaks for which at least one farm was affected by BTV within each 5 km grid square. Results are based on 100 replicates of the model with parameters sampled from the joint posterior distribution.

For the model in which vector dispersal is described as a diffusion process, the predicted number of newly infected holdings each week reaches its peak about seven weeks after the initial incursion (Fig 1A), preceding the peak in newly confirmed clinical farms by one or two weeks (Fig 1B). Moreover, there are substantially more infected farms than confirmed clinical farms, indicating a high level of under-ascertainment. The observed number of newly confirmed clinical farms each week lies close to the posterior mean for most weeks (Fig 1B), while the observed cumulative number of confirmed clinical farms in each county lies in the 95% prediction interval for all counties, except Essex (Fig 1C). In addition, the model predicts spread of BTV into areas where reported clinical farms were not confirmed in only a small proportion (2.7%) of simulations and, in each instance, only a single case (Fig 1C and 1F). The observed number of infected farms detected by pre-movement testing or by targeted surveillance lies within the 95% prediction intervals for the model (Fig 1D), while the posterior mean for the within-farm prevalence for cattle herds matches that observed (Fig 1E).

The predicted dynamics for the four kernel models are similar to those for the diffusion model (S1–S4 Figs; cf. Fig 1). However, all four kernel models predict more extensive spatial spread of BTV than the diffusion model. This has the consequence that they are less able to capture the number of infected holdings detected through targeted surveillance around the first two IPs, but are better able to account for the number of confirmed clinical farms in Essex. The kernel models also more frequently (>10% of simulations for each model) predict clinical cases in areas in which no cases were reported. In addition, the predictions of the kernel models are more variable than those for the diffusion model.

The marginal posterior distributions for the parameters in each model are plotted in S5 and S6 Figs and are summarised in S5 and S6 Tables. Summary statistics for the within-farm parameters are provided only for the model in which vector dispersal was described as a diffusion process (S6 Table). However, the posterior distributions did not differ greatly for most parameters (S6 Fig), except for the probability of transmission from host to vector (*β*), which was higher for the kernel models compared with the diffusion model (posterior mean: 0.05 vs 0.02).

### Quantifying transmission routes for BTV

**Fig 2:**
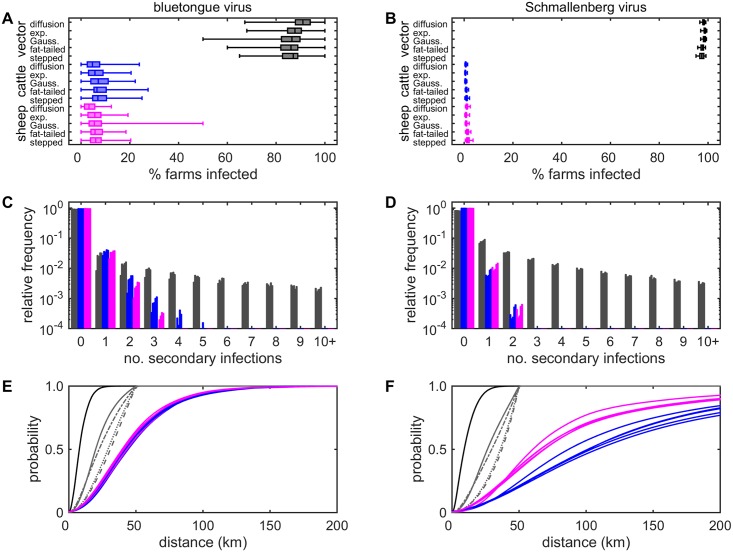
Quantifying the importance of transmission routes between farms for Bluetongue Virus (BTV) and Schmallenberg Virus (SBV). (A,B) Proportion (%) of farms which are infected via dispersal of infected vectors (grey) or movement of infected cattle (blue) or sheep (magenta). Box-and-whisker plots show the posterior median (horizontal line), interquartile range (box) and 2.5th and 97.5th percentiles (whiskers). (C,D) Frequency distribution for infected farms generating a number of secondary infections via vector dispersal (grey), movement of infected cattle (blue) or movement of infected sheep (magenta). (E,F) Cumulative density function for the distance between source and recipient farms when transmission occurs via vector dispersal (black or grey lines), movement of infected cattle (blue) or movement of infected sheep (magenta). Vector dispersal models are diffusion (black line), exponential kernel (exp.; dashed grey line), Gaussian kernel (Gauss.; dotted grey line), fat-tailed kernel (dash-dotted grey line) and stepped kernel (solid grey line). Results are based on 100 replicates of the model with parameters sampled from the joint posterior distribution.

All the models predict that transmission of BTV between farms occurs predominantly through dispersal of infected vectors. In simulated outbreaks, the median proportion of farms infected via dispersal of infected vectors was 86–91%, depending on the model for vector dispersal (Fig 2A). This compares with the median proportion of farms infected via movement of cattle and sheep of 5–7% and 3–6%, respectively (Fig 2A). A similar pattern is seen in the number of secondary infections via the three routes, in which most secondary infections are generated by vector dispersal (Fig 2C). In addition, the number of secondary infections per farm by vector dispersal is over-dispersed, with the majority of transmission attributable to a small number of farms (Fig 2C). The mean number of secondary infections for this route was around 1.3 for the diffusion model and around 0.9 for the kernel models, while the dispersion parameter was around 0.05 for all models (Table 2). By contrast, the mean number of secondary infections per farm arising by the movement of infected cattle or sheep was around 0.05 (Table 2).

**Table 2:** Estimated mean and dispersion parameters (mean and 95% credible interval) for the number of secondary infections per infected farm for different routes of transmission of bluetongue virus and Schmallenberg virus between farms.

model	bluetongue virus		Schmallenberg virus	
	mean	dispersion parameter	mean	dispersion parameter
*diffusion model*				
vector dispersal	1.27 (0.28, 2.13)	0.03 (0.01, 0.09)	1.98 (1.47, 2.93)	0.07 (0.04, 0.13)
cattle movement	0.04 (0.01, 0.12)	0.25 (0.01, 0.78)	0.01 (0.002, 0.01)	0.08 (0.04, 0.21)
sheep movement	0.04 (0.01, 0.13)	0.34 (0.02, 0.88)	0.01 (0.006, 0.02)	0.30 (0.10, 0.79)
*exponential kernel*				
vector dispersal	0.89 (0.13, 1.35)	0.06 (0.13, 1.35)	1.43 (1.26, 1.75)	0.07 (0.05, 0.10)
cattle movement	0.06 (0.02, 0.15)	0.26 (0.02, 1.20)	0.01 (0.002, 0.01)	0.10 (0.04, 0.45)
sheep movement	0.05 (0.02, 0.13)	0.46 (0.02, 2.69)	0.01 (0.003, 0.02)	0.28 (0.11, 0.63)
*Gaussian kernel*				
vector dispersal	0.89 (0.14, 1.31)	0.04 (0.01, 0.16)	1.46 (1.29, 1.78)	0.07 (0.05, 0.10)
cattle movement	0.06 (0.02, 0.16)	0.60 (0.02, 1.13)	0.006 (0.002, 0.01)	0.10 (0.04, 0.22)
sheep movement	0.05 (0.02, 0.13)	0.48 (0.02, 3.30)	0.01 (0.004, 0.02)	0.31 (0.07, 1.06)
*fat-tailed kernel*				
vector dispersal	0.89 (0.14, 1.23)	0.05 (0.01, 0.21)	1.41 (1.24, 1.67)	0.08 (0.06, 0.12)
cattle movement	0.07 (0.02, 0.17)	0.24 (0.02, 1.08)	0.01 (0.005, 0.02)	0.11 (0.04, 0.39)
sheep movement	0.05 (0.02, 0.14)	0.37 (0.03, 1.89)	0.01 (0.006, 0.03)	0.30 (0.11, 1.53)
*stepped kernel*				
vector dispersal	0.90 (0.14, 1.28)	0.05 (0.01, 0.17)	1.40 (1.18, 1.77)	0.08 (0.05, 0.13)
cattle movement	0.07 (0.02, 0.16)	0.21 (0.02, 0.90)	0.01 (0.004, 0.02)	0.10 (0.04, 0.25)
sheep movement	0.06 (0.02, 0.14)	0.33 (0.02, 1.14)	0.02 (0.006, 0.03)	0.29 (0.11, 0.72)

The distance over which transmission occurred was strongly dependent on the transmission route. Transmission via movement of infected livestock occurred over considerably longer distances than via dispersal of infected vectors and this was independent of the model used for vector dispersal (Fig 2E). When transmission was via movement of infected livestock, the mean distance between source and recipient farms was around 50 km, with 99% of transmission occurring within around 150 km for both cattle and sheep. When transmission was via dispersal of infected vectors, the distances between source and recipient farms depended critically on the model for vector dispersal (Fig 2E). The median distance (distance within which 99% of transmission occurred) was 7.8 (25.2) km for the diffusion, 30.9 (49.6) km for the exponential kernel, 29.1 (49.5) km for the Gaussian kernel, 22.5 (49.3) km for the fat-tailed kernel and 19.9 (48.6) km for the stepped kernel.

**Fig 3:**
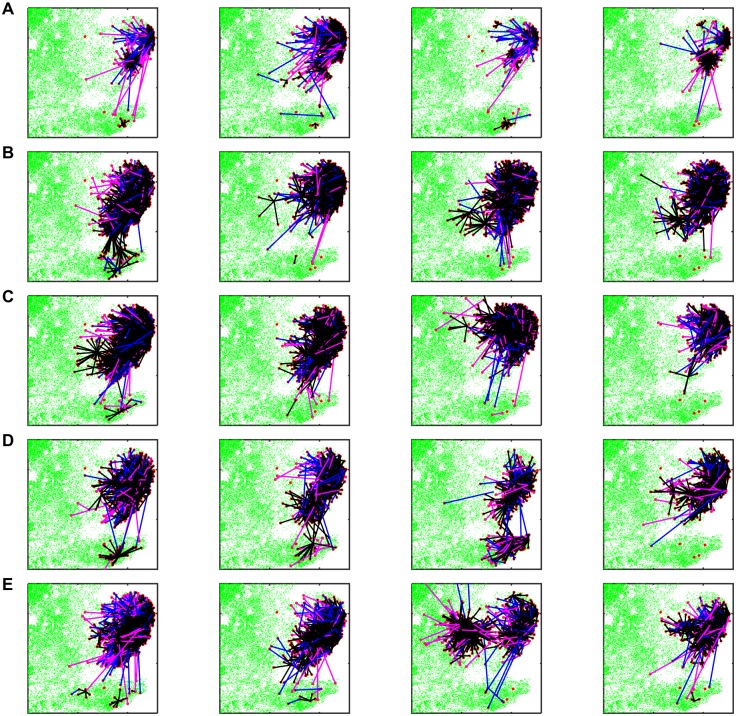
Transmission routes in simulated outbreaks of bluetongue virus in 2007 in Great Britain. Maps show transmission routes assuming different models for vector dispersal: (A) diffusion; (B) exponential kernel; (C) Gaussian kernel; (D) fat-tailed kernel; or (E) stepped kernel. In each plot uninfected farms are shown as green dots and infected farms are shown as red dots. Unconnected red dots are seed farms which did not infect other farms. Lines indicate transmission via dispersal of infected vectors (black), movement of infected cattle (blue) or movement of infected sheep (magenta). Results are presented for four selected replicates of the each model with parameters sampled from the joint posterior distribution.

The characteristics of each transmission route (frequent, but shorter range for vector dispersal; less frequent, but longer range for cattle and sheep movements) and the differences between models in vector dispersal distances are demonstrated in maps showing which farm infected which in the simulated outbreaks (Fig 3).

The sensitivity of the importance of the transmission routes to the time of incursion and temperature data was assessed for each model of vector dispersal (S7 and S8 Figs). The proportion of farms infected via each route was not substantially influenced by either the time of incursion or the temperature data used. Both the number of secondary infections per infected farm and the distance over which BTV spread via livestock movements were higher for incursions earlier in the year. Furthermore, the number of secondary infections per infected farm was higher when using the 2006 temperature data (a warmer year) compared with 2007 data (a cooler year).

### Comparison of BTV and SBV

The model predicts much larger outbreaks for SBV compared with BTV, in terms of both the number of infected farms and spatial spread (S9 Fig; cf. Fig 1). Furthermore, the proportion of transmission between farms via dispersal of infected vectors is higher for SBV than for BTV (Fig 2B). In simulated outbreaks the median proportion of farms infected via vector dispersal is 98% and this is independent of the model of vector dispersal. This compares with 1% each for transmission via movement of infected cattle and sheep. This difference in the importance of the transmission routes was reflected in the number of secondary infections per infected farm, which was higher for vector dispersal for SBV than for BTV, but which was lower for cattle and sheep movements (Fig 2D; cf. Fig 2C). For SBV, the mean number of secondary infections per infected farm was around 2.0 for the diffusion model and around 1.5 for the kernel models, while the dispersion parameter was around 0.07 for all models (Table 2). The mean number of secondary infections via livestock movements was 0.01 for both cattle and sheep (Table 2). The distance over which SBV spread via livestock movements was greater than for BTV (Fig 2F), but this is a consequence of the movement restrictions in place during the BTV outbreak. Finally, the importance of the transmission routes for SBV was not greatly sensitive to the time of incursion (S10 Fig).

### Impact of movement restrictions on spread

**Fig 4:**
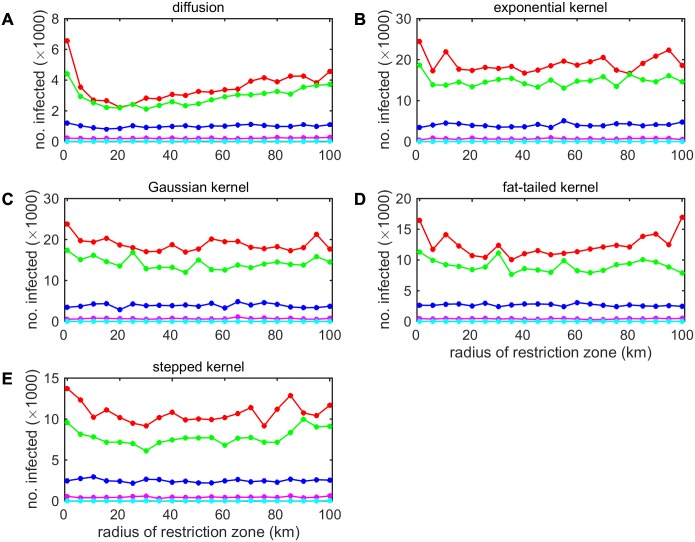
Impact of the radius of the movement restriction zone on the size of outbreaks of bluetongue virus in Great Britain. Each figure shows the mean cumulative number of infected farms (circles and lines) for simulations assuming different models for vector dispersal: (A) diffusion; (B) exponential kernel; (C) Gaussian kernel; (D) fat-tailed kernel; or (E) stepped kernel. Colour indicates the time of incursion: 1 May (red), 1 June (green), 1 July (blue), 1 August (magenta) or 1 September (cyan). For each scenario 100 replicates of the model were simulated using movement data for 2006 and temperature data for 2007.

Movement restrictions (in this case applied in a circular zone around known IPs) can potentially reduce the size of a BTV outbreak, but whether or not they are predicted to do so depends critically on assumptions about vector dispersal (Fig 4). When vector dispersal is described by a diffusion process, movement restrictions reduce the size of an outbreak and, furthermore, there is an optimal radius for the MRZ of approximately 20 km (Fig 4A). When vector dispersal is described by a fat-tailed kernel, there is also some evidence for an impact of movement restrictions on outbreak size, but in this case the optimal MRZ radius is around 35–40 km (Fig 4D). However, when vector dispersal is described by an exponential, Gaussian or stepped kernel, there is no evidence for an impact of movement restrictions (Fig 4B, 4C and 4E). In addition, movement restrictions do not substantially reduce outbreak size if the incursion occurs later in the year (August or September) and this conclusion is independent of assumptions about vector dispersal (Fig 4). Similar results are also obtained if temperature data for 2006 are used (S11 Fig) instead of for 2007 (Fig 4).

Whether or not movement restrictions are predicted to reduce the size of an SBV outbreak also depends critically on assumptions about vector dispersal (S12 Fig). Using the model describing vector dispersal as a diffusion process, movement restrictions were predicted to have a substantial impact on outbreak size and to a much greater extent than for BTV (S12 Fig; cf. Fig 4). By contrast, movement restrictions were predicted to have no impact on outbreak size for any of the kernel models (S12 Fig).

## Discussion

Initial modelling studies for BTV-8 in northern Europe used kernel- or wave-based approaches to explore spread, implicitly incorporating all modes of transmission in a single description [18,35,39]. Subsequently, models were developed which separate animal and vector movements [40–42], but these were not fitted to outbreak data nor did they quantify the relative importance of the two transmission routes.

Here we have developed a model framework that allows us to disentangle and quantify the roles played by livestock movements and vector dispersal in the transmission of two *Culicoides*-borne viruses. Our results show that dispersal of infected vectors accounts for the majority (around 90%) of spread of BTV between farms and an even higher proportion (98%) of spread of SBV between farms (Fig 2A and 2B). We are able to attribute spread to each route because of the detailed, independent data available to describe spread via movement of infected livestock. If this were not the case, it would be more challenging to estimate the relative contribution of each route, because a decrease in transmission due to one route could be compensated for by an increase in transmission due to another.

One previous study has quantified the role of vector dispersal in the spread of BTV-8 in northwest Europe in 2006 [31]. The authors could explain infection onset for 94% of reported BTV-infected farms based on wind and midge flight activity. As they did not consider livestock movements, this represents an upper bound on the proportion of farms infected via vector dispersal, but is consistent with our estimate for the BTV-8 outbreak in GB in 2007 (Fig 2A). Similar methods were subsequently applied to quantify the role of vector dispersal in the spread of SBV in northwest Europe in 2011 [32]. In contrast with BTV, the authors could explain infection onset for only 70% of reported SBV-infected farms based on midge flight activity, which is markedly lower than our estimate of 98% (Fig 2B). This discrepancy is likely to be a consequence of under-ascertainment of SBV-infected farms. This results in a greater distance between infected farms, making it more difficult for the vector-only approach to explain SBV transmission [32].

The number of secondary infections arising through each route also emphasises the major role played by vector dispersal in the transmission of BTV and SBV between farms compared with livestock movements (Fig 2C and 2D). In particular, the mean number of secondary infections via both cattle and sheep movements was estimated to be substantially below one for both viruses (Table 2), indicating that these routes alone cannot sustain transmission (cf. [41]). By contrast, the mean number of secondary infections via vector dispersal was above one for both BTV and SBV, indicating transmission can be sustained by this route. However, the number of secondary infections is over-dispersed, so that a small proportion of farms account for the majority of transmission: an example of the 80/20 rule [43].

In the model, it is the larger farms which account for most of the transmission via dispersal of infected vectors. This is primarily a consequence of our assumption that the number of vectors is proportional to the number of livestock on a farm (though the constant of proportionality does vary amongst farms). Few studies have investigated the relationship between vector abundance and host numbers. One recent study found that *Culicoides* abundance was higher at trap locations with a high density of cattle in the locality [24]. Another study suggested that catches in light traps increase linearly with sheep numbers, at least for small host numbers [44]. Although these results do not allow robust generalizations, the findings are compatible with the assumption of a constant vector-to-host ratio. In addition, the common alternative assumption is that the number of vectors is independent of host numbers, but this results in the conclusion that outbreaks are more likely on smaller farms because they will have higher vector-to-host ratios.

Both the proportion of transmission and the number of secondary infections via dispersal of infected vectors are independent of the model used for vector dispersal (Fig 2A–2D). This is not the case, however, for the distances between source and recipient farms, which differ markedly amongst the models (Fig 2E and 2F). The distances inferred in the present study of BTV-8 in GB using a diffusion model (median: 7.8 km; Fig 2E) are similar to those estimated for BTV-8 in northern Europe based on wind and midge flight activity (median: ~5 km; see [31], their Fig 3). These contrast with the considerably larger distances inferred using the kernel models (median for all models: ≥20 km; Fig 2E). Comparing the different models suggests that, while the diffusion model is able to capture the general pattern of local-scale spread, it is not able to capture the relatively infrequent longer-range dispersal events (see, e.g. [30]). By contrast, the kernel models can predict longer-range jumps, but at the expense of missing the detail of local-scale spread. However, given the under-ascertainment of infected holdings and the spatial resolution of the data, it will be difficult to infer a more robust model for vector dispersal from the 2007 GB outbreak. Moreover, the challenges associated with studying *Culicoides* biting midges in the field [45–47] make it difficult to estimate dispersal patterns empirically, especially over longer distances.

The lower proportion of transmission attributed to movement of infected livestock for SBV compared with BTV (2% vs 10%; Fig 2A and 2B) can be accounted for by two key differences between the viruses. First, vector competence (i.e. the probability of transmission from host to vector) is much higher for SBV (0.14; [19]) than for BTV (0.02; S6 Table). This means there is a higher prevalence of infectious vectors for SBV compared with BTV, increasing the importance of vector dispersal for SBV (see Eqs (5) and (6)). Second, the mean duration of viraemia is much longer for BTV (21 days; S6 Table) than for SBV (3–4 days; [19]). Consequently, there is a lower probability of moving an animal while it is infected for SBV than for BTV, reducing the importance of livestock movements for SBV.

Host movements may only account for a small proportion of transmission, but our results reinforce the important role that they play in the transmission of vector-borne diseases, particularly through the introduction of infection to new areas [3,4,48,49]. In the case of BTV and SBV, host (i.e. livestock) movements spread the virus over much longer distances than would be expected by vector dispersal (Fig 2E and 2F) and facilitate the establishment of new outbreaks away from existing foci (Fig 3).

While seldom practical for human movements, it is feasible to restrict livestock movements as part of disease control measures. The predicted impact of movement restrictions for both BTV and SBV depends critically on the model used for vector dispersal. In particular, there is a significant reduction in outbreak size (i.e. cumulative number of farms infected) only for the diffusion model (Fig 4; S11 and S12 Figs). Furthermore, the magnitude of the reduction resulting from movement restrictions is predicted to be much lower than alternative control measures, in particular, vaccination [50,51].

The difference in predicted effectiveness of movement restrictions amongst models reflects the distances over which dispersal of infected vectors occurs in each one (Fig 2E and 2F). When dispersal is primarily local, as is the case with the diffusion model, movement restrictions are effective because the virus is unlikely to escape the MRZ through vector dispersal into an area in which movements are allowed and, hence, can spread over longer distances. When vector dispersal occurs more frequently over longer distances, as is the case with the exponential and Gaussian kernels, infection is likely to escape any MRZ through vector dispersal alone. As a result, movement restrictions are predicted to be ineffective in this case.

When fitting the BTV model to outbreak data we have used summary or aggregated epidemiological measures, rather than more detailed data on location and timing of infected farms. For many outbreaks, including the 2007 BTV outbreak in GB, summary statistics are the only data available, which makes ABC a natural framework in which to implement epidemiological models [37,52]. Moreover, ABC methods facilitate integrating data from different surveillance sources (in the case of BTV, reported clinical farms, pre-movement testing and targeted surveillance), which helps overcome the limitations associated with individual sources (e.g. under-ascertainment of reported cases). This does, however, require a model relating disease occurrence and reporting to the underlying pattern of infection, which is not always straight-forward. Here, we have used a simple model (a fixed daily probability of reporting), which captures this acceptably for most areas in each of the models. Where there are discrepancies (e.g. for Essex in the diffusion model; Fig 1C), this could reflect differences in reporting behaviour between regions or changes in the probability of reporting over time. This is difficult to explore in detail, however, given the limited numbers of cases.

In this study we have demonstrated that both vector dispersal and host movements play important roles in transmission of vector-borne diseases of livestock, though for different reasons. Vector dispersal is the principal mode of spread between farms, while livestock movement is the principal means of introducing infection to new areas. However, the relative importance of the routes differs between viruses, even when they share the same vector species. This has practical implications for disease control and, in particular, movement restrictions, so that generic measures may not be effective.

## Supporting information

S1 Data Summary outbreak measures for bluetongue virus in Great Britain, 2007. (XLSX)

S1 Text Model for t ransmission via movement of infected livestock. (DOCX)

S2 Text Implementation of the approximate Bayesian computation sequential Monte Carlo scheme. (DOCX)

S1 Fig Observed and predicted spread of bluetongue virus serotype 8 in Great Britain in 2007 for the model in which vector dispersal is described by an exponential kernel. Panels A-F are the same as for Fig 1 in the main paper and results are based on 1000 replicates of the model with parameters sampled from the joint posterior distribution.
(TIF)

S2 Fig Observed and predicted spread of bluetongue virus serotype 8 in Great Britain in 2007 for the model in which vector dispersal is described by a Gaussian kernel. Panels A-F are the same as for Fig 1 in the main paper and results are based on 1000 replicates of the model with parameters sampled from the joint posterior distribution.
(TIF)

S3 Fig Observed and predicted spread of bluetongue virus serotype 8 in Great Britain in 2007 for the model in which vector dispersal is described by a fat-tailed kernel. Panels A-F are the same as for Fig 1 in the main paper and results are based on 1000 replicates of the model with parameters sampled from the joint posterior distribution.
(TIF)

S4 Fig Observed and predicted spread of bluetongue virus serotype 8 in Great Britain in 2007 for the model in which vector dispersal is described by a stepped kernel. Panels A-F are the same as for Fig 1 in the main paper and results are based on 1000 replicates of the model with parameters sampled from the joint posterior distribution.
(TIF)

S5 Fig Marginal posterior distributions for parameters in the diffusion and kernel models for the transmission of bluetongue virus between farms. (A) Vector transmission parameter, *γ* for each model. (B) Vector diffusion coefficient, *D*. (C). Exponential kernel parameter (α). (D) Gaussian kernel parameter (α). (E) Fat-tailed kernel distance scaling (*d*_0_). (F) Fat-tailed kernel power (α). (G) Stepped kernel distance parameter (*d*_0_). (H) Stepped kernel power (α). In each plot the posterior density for the parameter is shown for the diffusion model (black), exponential kernel (red), Gaussian kernel (green), fat-tailed kernel (blue) and stepped kernel (magenta); the prior distribution is shown in cyan.
(TIF)

S6 Fig Marginal posterior distributions for the parameters in the model for the transmission of bluetongue virus within a farm. In each plot the posterior density for the parameter is shown for the diffusion model (black), exponential kernel (red), Gaussian kernel (green), fat-tailed kernel (blue) and stepped kernel (magenta); the prior distribution is shown in cyan.
(TIF)

S7 Fig Quantifying the importance of transmission routes for bluetongue virus between farms for different times of incursion assuming 2006 temperatures. Left-hand column: proportion (%) of farms which are infected via dispersal of infected vectors (grey) or movement of infected cattle (blue) or sheep (magenta). Box-and-whisker plots show the posterior median (horizontal line), interquartile range (box) and 2.5th and 97.5th percentiles (whiskers). Middle column: frequency distribution for infected farms generating a number of secondary infections via vector dispersal (grey), movement of infected cattle (blue) or movement of infected sheep (magenta). Bars are for incursions in (from left to right) May, June, July, August or September. Right-hand column: cumulative density function for the distance between source and recipient farms when transmission occurs via vector dispersal (black), movement of infected cattle (blue) or movement of infected sheep (magenta). Lines are for incursions in May (right-most solid), June (dashed), July (dotted), August (dot-dashed) or September (left-most solid). Results are based on 100 replicates of the model identified in the panel title with parameters sampled from the joint posterior distribution.
(TIF)

S8 Fig Quantifying the importance of transmission routes for bluetongue virus between farms for different times of incursion assuming 2007 temperatures. Panels are the same as in S7 Fig.
(TIF)

S9 Fig Predicted spread of Schmallenberg virus in Great Britain in 2011. Simulated outbreaks are shown for the model in which vector dispersal is described by (A) a diffusion process, (B) an exponential kernel, (C) a Gaussian kernel, (D) a fat-tailed kernel or (E) a stepped kernel. The left-hand column shows the mean (circles) and 95% prediction intervals for the weekly incidence (number of newly infected farms). The right-hand column shows the predicted spatial spread of SBV. The map shows the cumulative probability of infection (see scale bar) expressed as the proportion of simulated outbreaks for which at least one farm was affected by SBV within each 5 km grid square. Results are based on 100 replicates of each model with parameters sampled from the joint posterior distribution.
(TIF)

S10 Fig Quantifying the importance of transmission routes for Schmallenberg virus between farms for different times of incursion and temperature data. (A,B) Proportion (%) of farms which are infected via dispersal of infected vectors (grey) or movement of infected cattle (blue) or sheep (magenta). Box-and-whisker plots show the posterior median (horizontal line), interquartile range (box) and 2.5th and 97.5th percentiles (whiskers). (C,D) Frequency distribution for infected farms generating a number of secondary infections via vector dispersal (grey), movement of infected cattle (blue) or movement of infected sheep (magenta). Bars are for incursions in (from left to right) May, June, July, August or September. (E,F) Cumulative density function for the distance between source and recipient farms when transmission occurs via vector dispersal (black), movement of infected cattle (blue) or movement of infected sheep (magenta). Lines are for incursions in May (right-most solid), June (dashed), July (dotted), August (dot-dashed) or September (left-most solid). Results are based on 100 replicates of the diffusion model with parameters sampled from the joint posterior distribution using temperature data for (A,C,E) 2006 or (B,D,F) 2007.
(TIF)

S11 Fig Impact of the radius of the movement restriction zone on the size of outbreaks of bluetongue virus in Great Britain. Each figure shows the mean cumulative number of infected farms (circles and lines) for simulations assuming different models for vector dispersal: (A) diffusion; (B) exponential kernel; (C) Gaussian kernel; (D) fat-tailed kernel; or (E) stepped kernel. Colour indicates the time of incursion: 1 May (red), 1 June (green), 1 July (blue), 1 August (magenta) or 1 September (cyan). For each scenario 100 replicates of the model were simulated using movement data for 2006 and temperature data for 2006.
(TIF)

S12 Fig Impact of the radius of the movement restriction zone on the size of outbreaks of Schmallenberg virus in Great Britain. Each figure shows the mean cumulative number of infected farms (circles and lines) for simulations assuming different models for vector dispersal: (A) diffusion; (B) exponential kernel; (C) Gaussian kernel; (D) fat-tailed kernel; or (E) stepped kernel. Colour indicates the time of incursion: 1 May (red), 1 June (green), 1 July (blue), 1 August (magenta) or 1 September (cyan). For each scenario 100 replicates of the model were simulated using movement data for 2006 and temperature data for 2007.
(TIF)

S1 Table Parameters in the model for the transmission of bluetongue virus within a farm. (DOCX)

S2 Table Transitions, probabilities and population sizes in the model for the transmission of bluetongue virus within a farm. (DOCX)

S3 Table Parameters in the logistic regression models for the probability of a farm moving (off move) and receiving (on move) cattle and sheep. (DOCX)

S4 Table Probability distribution for the number of batches of cattle or sheep moved off a farm which moves any livestock on a given day. (DOCX)

S5 Table Posterior mean and 95% credible intervals for parameters in the diffusion and kernel models for the transmission of bluetongue virus between farms. (DOCX)

S6 Table Posterior mean and 95% credible intervals for parameters in the model for the transmission of bluetongue virus within farms. (DOCX)
