# Supplementary material for: Quantifying the roles of host movement and vector dispersal in the transmission of vector-borne diseases of livestock
Source: PLoS Comput Biol. 2017 Apr 3;13(4):e1005470. doi: 10.1371/journal.pcbi.1005470 (PMC5393902; doi:10.1371/journal.pcbi.1005470)
Supplement: S1 Text — (DOCX) [file pcbi.1005470.s002.docx]

**S1 Text. Model for transmission via movement of infected livestock**

Here we provide details of the distributions and parameters required for each step when modelling the transmission of bluetongue virus between farms via movement of infected livestock.

**Step 1: Number of off-moves.** The probability that a farm moves a batch of animals off the farm (i.e. makes an off-move), *p_OFF_*, is given by,

$$\log_{e}\left(\frac{p_{OFF}}{1-p_{OFF}}\right)=\alpha_{0}+\alpha_{1}H+\alpha_{2}(m),$$

where *α*_0_ and *α*_1_ are constants, *H* is the herd or flock size and *α*_2_(*m*) is the log odds ratio for selling animals in month *m* (reflecting seasonality in animal movements) (S3 Table). If a farm does make an off-move, the number of off-moves made that day is drawn from a multinomial distribution, which was computed empirically from the observed number of off-moves (S4 Table).

**Step 2: Batch size distribution.** For each off-move the batch size (*B*) was determined by sampling from a negative binomial distribution, that is,

$$\Pr(B=b)=\frac{\Gamma(k+b)}{b!\Gamma(k)}\left(\frac{k}{k+\mu}\right)^{k}\left(\frac{\mu}{k+\mu}\right)^{b},$$

where *µ* and *k* are the mean and dispersion parameter, respectively. These were estimated by fitting the distribution to the observed batch sizes (cattle: *µ*=2.02, *k*=0.33; sheep: *µ*=38.2, *k*=0.66). The number of infected animals in the batch (*J*) are drawn from a hypergeometric distribution, so that,

$$\Pr(J=j)=\frac{\left(\begin{matrix} Y \\ j \end{matrix}\right)\left(\begin{matrix} H-Y \\ B-j \end{matrix}\right)}{\left(\begin{matrix} H \\ B \end{matrix}\right)},$$

where *H* is the herd or flock size and *Y* is the number of infected animals in the herd or flock (determined from the simulated within-farm outbreak).

**Step 3a: Selecting a county for an on-move.** The county for each on-move was selected based on the relative frequency of movements from the county in which the affected flock is located to all counties.

**Step 3b: Probability of an on-move.** The probability that a farm makes an on-move (i.e. buys in animals), *p_ON_*, was given by,

$$\log_{e}\left(\frac{p_{ON}}{1-p_{ON}}\right)=\beta_{0}+\beta_{1}N+\beta_{2}(m),$$

where *β*_0_ and *β*_1_ are constants, *N* is the herd or flock size and *β*_2_(*m*) is the log odds ratio for buying-in animals in month *m* (reflecting seasonality in animal movements) (S3 Table).
