# Supplementary material for: Quantifying the roles of host movement and vector dispersal in the transmission of vector-borne diseases of livestock
Source: PLoS Comput Biol. 2017 Apr 3;13(4):e1005470. doi: 10.1371/journal.pcbi.1005470 (PMC5393902; doi:10.1371/journal.pcbi.1005470)
Supplement: S2 Text — (DOCX) [file pcbi.1005470.s003.docx]

**S2 Text. Implementation of the approximate Bayesian computation sequential Monte Carlo scheme.**

Here we provide details of the implementation of the approximate Bayesian computation (ABC) sequential Monte Carlo (SMC) scheme used to estimate parameters in the BTV model.

The ABC-SMC scheme is implemented as the following sequence of steps [1,2]:

1. Start at round *t*=1. Initialise the tolerance for the goodness-of-fit metric, *ε*_1_, based on practice simulations.
2. Generate a particle (i.e. set of parameters), *θ_i_*_,_*_t_*:
   1. if *t*=1, sample from the joint prior density, *π*(*θ*);
   2. if *t*>1, sample the particles with the weights *w_j_*_,_*_t_*_-1_ generated during the previous round; perturb the particle by adding a Uniform(-0.1ξ*_t_*_-1_,0.1ξ*_t_*_-1_) variate to each parameter, where ξ*_t_*_-1_ is the range of the marginal distribution for the parameter in the previous round [3].
3. Simulate the model with the particle and calculate the summary outbreak measures.
4. Compare the simulated and observed summary outbreak measures using the goodness-of-fit metric, *M* (defined by equation (6) in the main paper). If *M*<*ε_t_*, accept the particle; otherwise (i.e. if *M*≥*ε_t_*), go back to step (b).
5. Calculate the weight for the particle,
6. if *t*=1, *w_i_*_,_*_t_*=1;
7. if *t*>1, the weight is given by,

$$w_{i,t}=\frac{\pi(\theta_{i,t})}{\sum_{j=1}^{N} w_{j,t-1}K(\theta_{i,t}|\theta_{j,t-1})},$$

where *π*(*θ_i,t_*) is the prior probability of the particle, *w_j_*_,_*_t_*_-1_ is the weight of particle *j* in the previous round and *K*(*θ_i,t_*|*θ_j,t_*_-1_) is the probability of moving from particle *j* to particle *i* (i.e. the perturbation kernel; see step (b)(ii)).

1. Repeat steps (b)-(e) until 10,000 particles have been accepted.
2. Normalise the particle weights, so that they sum to one.
3. Calculate a new tolerance from the median value of the *M*s for the round [3].
4. Increase the round number to *t*+1 and go to step (b).

Convergence of the posterior distributions was monitored by visual inspection of the outputs (posterior and outbreak measures) from consecutive SMC rounds. The acceptance tolerance (*ε_t_*) was set to 200 on the first round. For each model for vector dispersal (see main paper), convergence to the posterior distribution was achieved after 15-20 SMC rounds, with a final tolerance of around 20.

**References for S2 Text**

1. Toni T, Welch D, Strelowa N, Ipsen A, Stumpf MPH. Approximate Bayesian computation scheme for parameter inference and model selection in dynamical systems. J R Soc Interface 2009;6: 187-202.
2. McKinley T, Cook AR, Deardon R. Inference in epidemic models without likelihoods. Int J Biostat. 2009;5: 24.
3. Conlan AJK, McKinley TJ, Karolemeas K, Brooks Pollock E, Goodchild AV, Mitchell AP, Birch CPD, Clifton-Hadley RS, Wood JLN. Estimating the hidden burden of bovine tuberculosis in Great Britain. PLoS Comp Biol. 2012;8: e1002730.
