# Supplementary figures and images for: Quantifying the roles of host movement and vector dispersal in the transmission of vector-borne diseases of livestock
Source: PLoS Comput Biol. 2017 Apr 3;13(4):e1005470. doi: 10.1371/journal.pcbi.1005470 (PMC5393902; doi:10.1371/journal.pcbi.1005470)

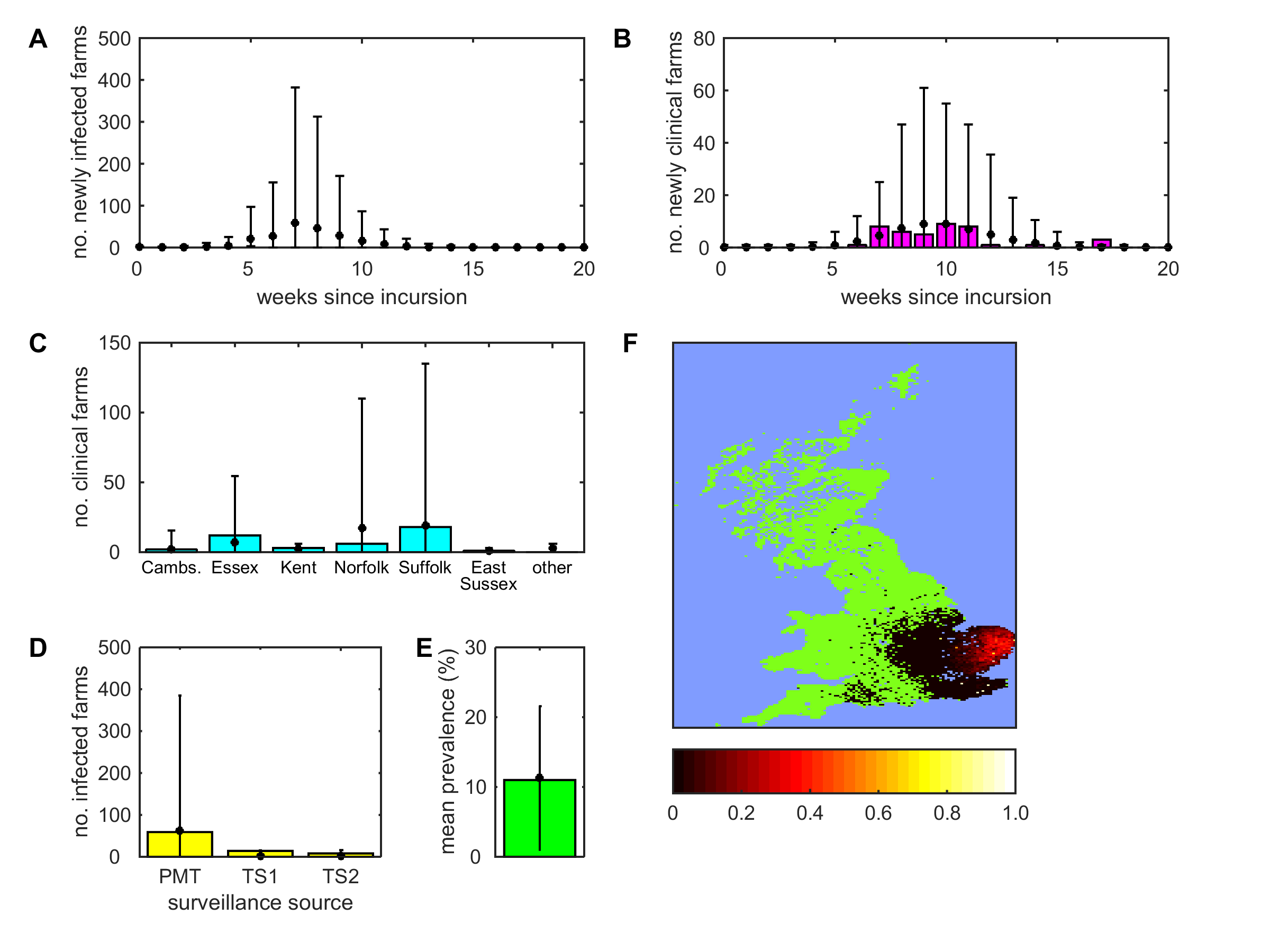

Supplement: S1 Fig — Panels A-F are the same as for Fig 1 in the main paper and results are based on 1000 replicates of the model with parameters sampled from the joint posterior distribution. (TIF) [file pcbi.1005470.s004.tif]

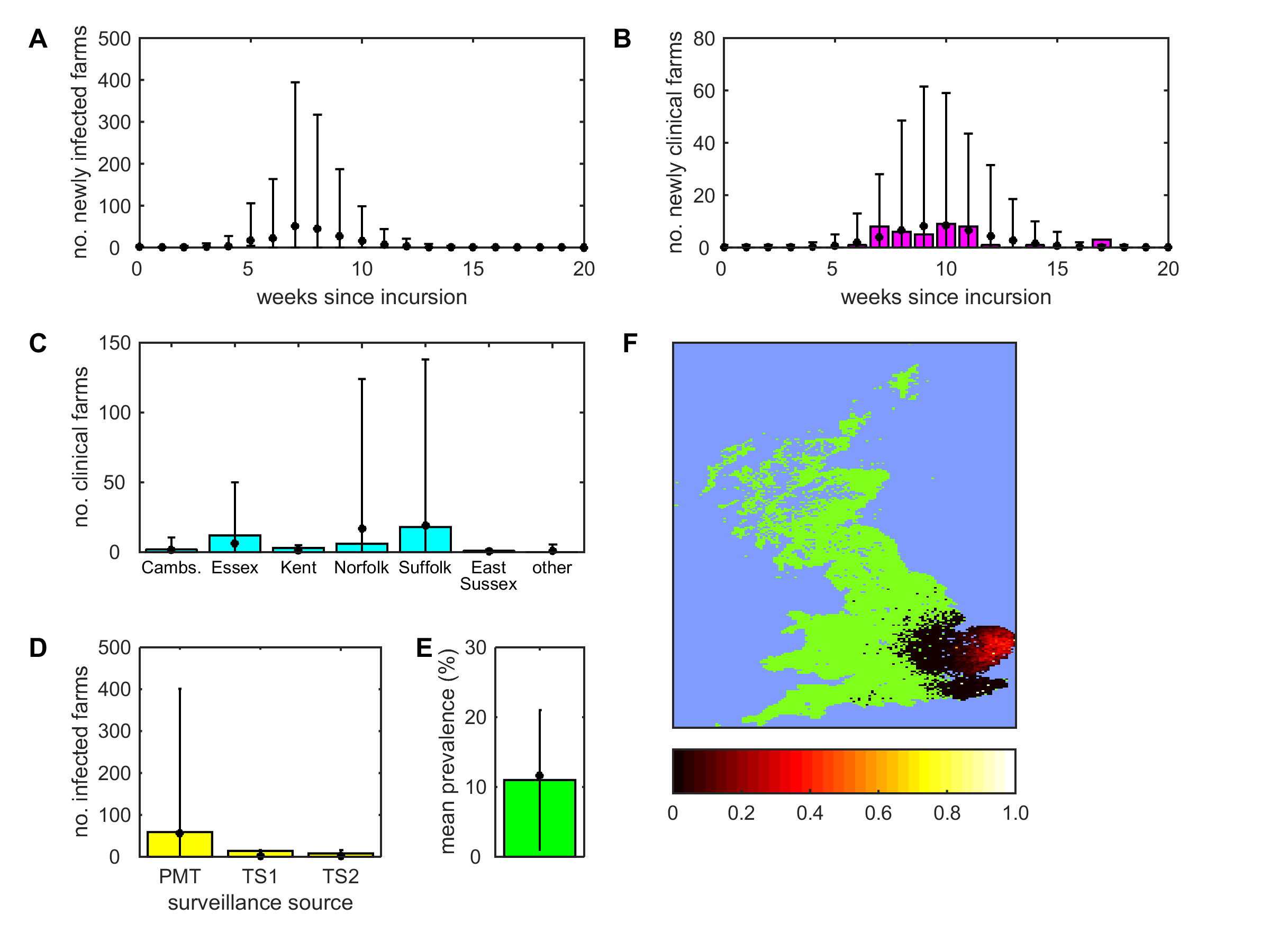

Supplement: S2 Fig — Panels A-F are the same as for Fig 1 in the main paper and results are based on 1000 replicates of the model with parameters sampled from the joint posterior distribution. (TIF) [file pcbi.1005470.s005.tif]

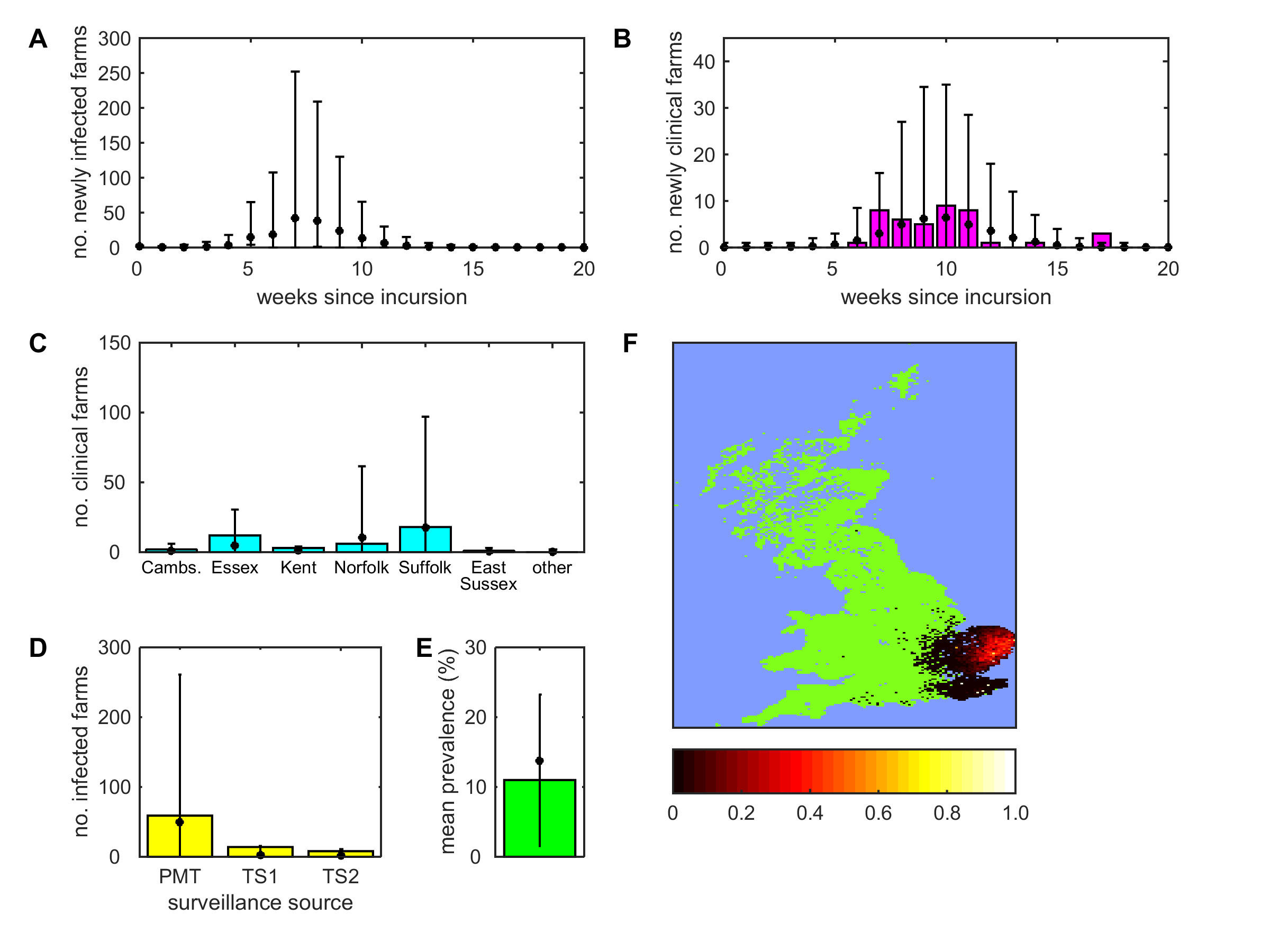

Supplement: S3 Fig — Panels A-F are the same as for Fig 1 in the main paper and results are based on 1000 replicates of the model with parameters sampled from the joint posterior distribution. (TIF) [file pcbi.1005470.s006.tif]

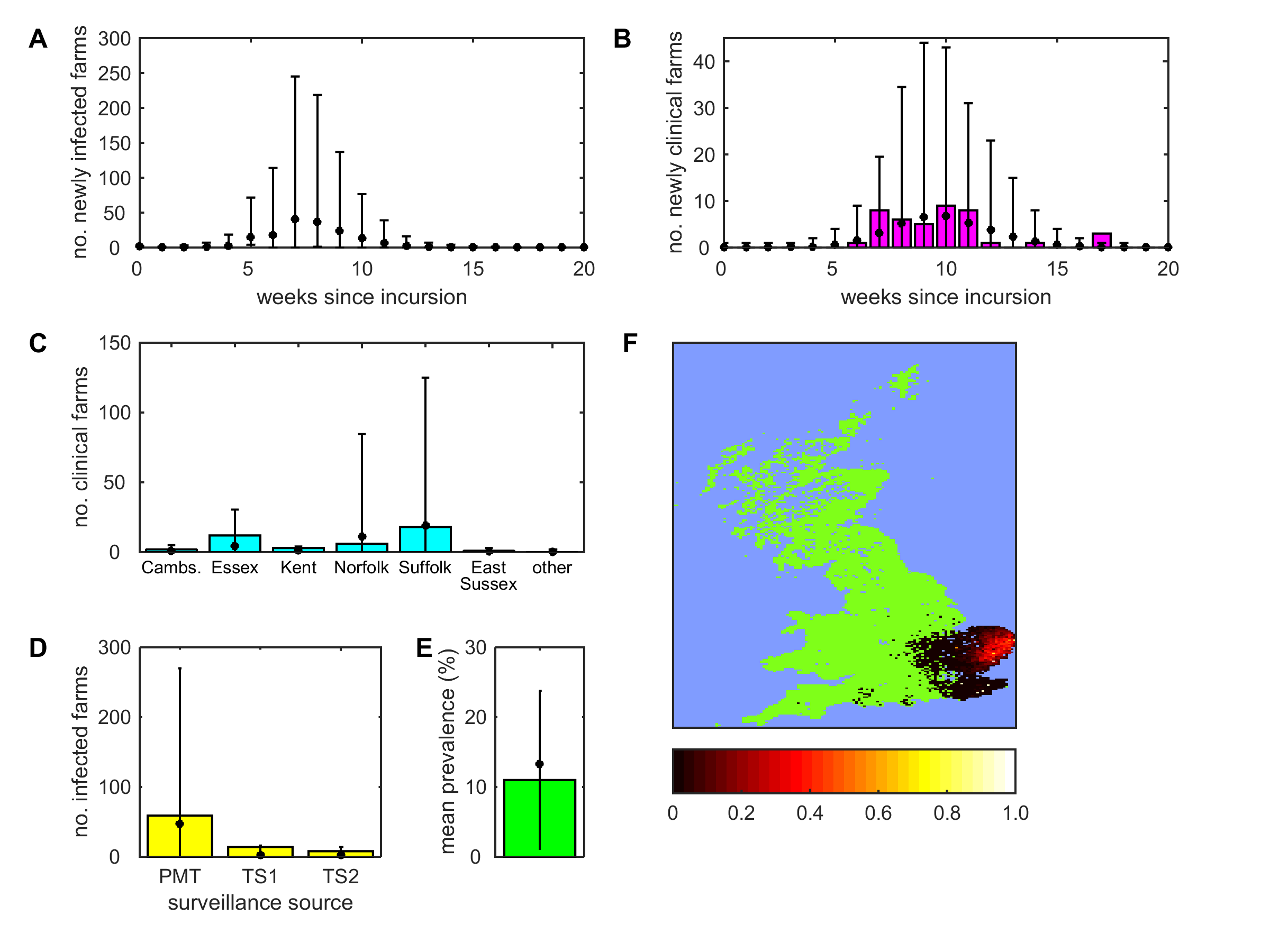

Supplement: S4 Fig — Panels A-F are the same as for Fig 1 in the main paper and results are based on 1000 replicates of the model with parameters sampled from the joint posterior distribution. (TIF) [file pcbi.1005470.s007.tif]

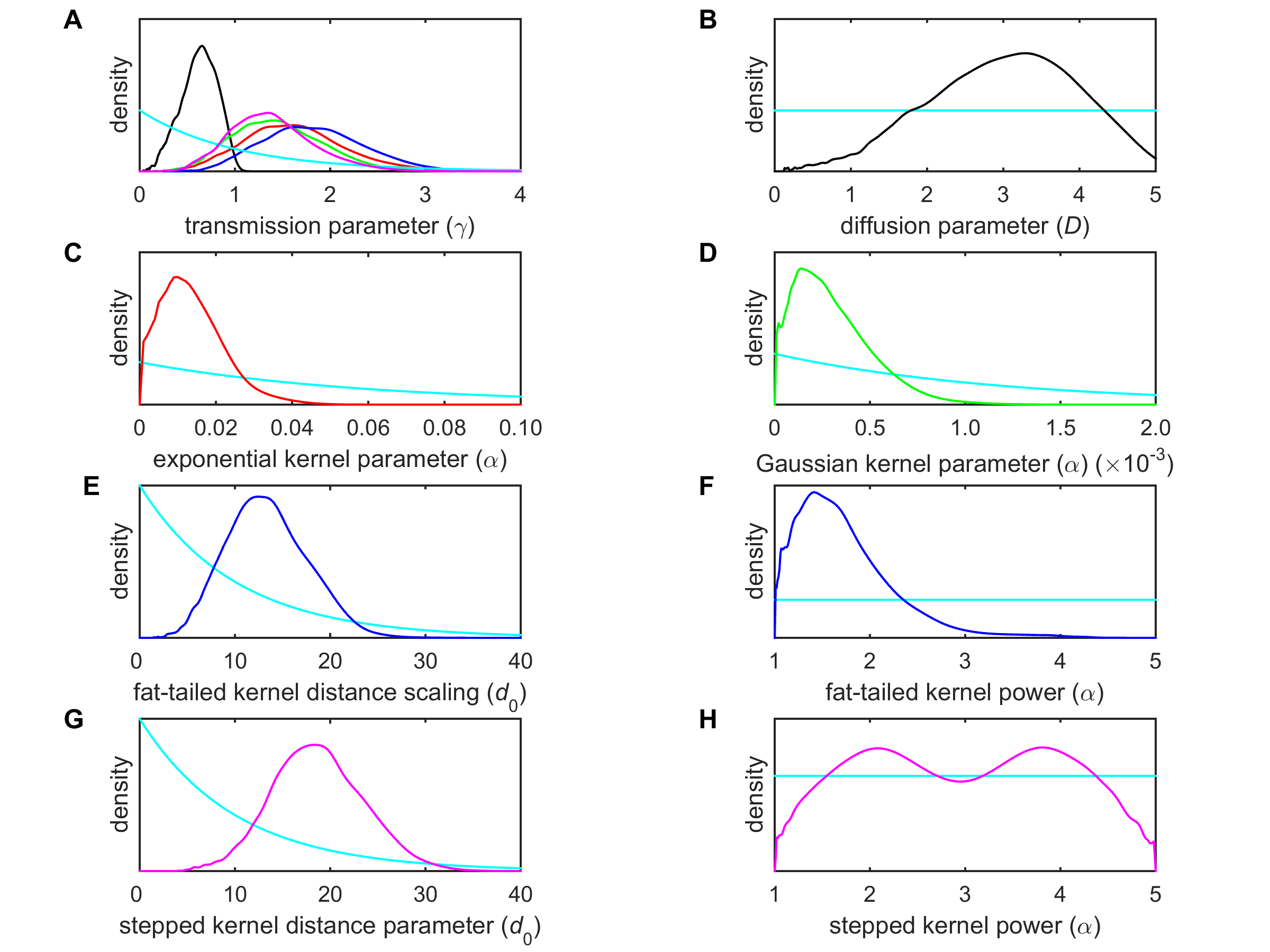

Supplement: S5 Fig — (A) Vector transmission parameter, γ for each model. (B) Vector diffusion coefficient, D. (C). Exponential kernel parameter (α). (D) Gaussian kernel parameter (α). (E) Fat-tailed kernel distance scaling (d0). (F) Fat-tailed kernel power (α). (G) Stepped kernel distance parameter (d0). (H) Stepped kernel power (α). In each plot the posterior density for the parameter is shown for the diffusion model (black), exponential kernel (red), Gaussian kernel (green), fat-tailed kernel (blue) and stepped kernel (magenta); the prior distribution is shown in cyan. (TIF) [file pcbi.1005470.s008.tif]

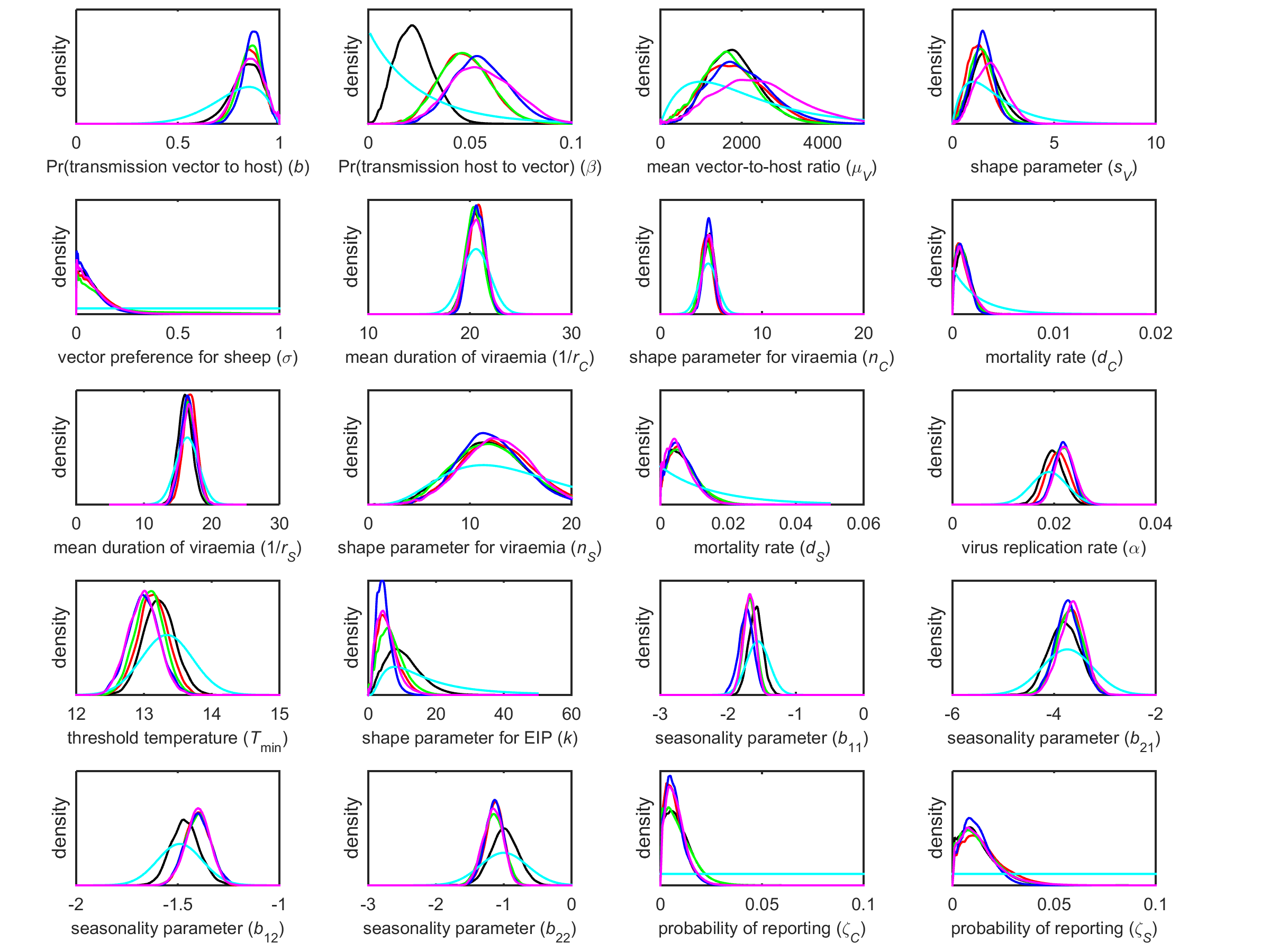

Supplement: S6 Fig — In each plot the posterior density for the parameter is shown for the diffusion model (black), exponential kernel (red), Gaussian kernel (green), fat-tailed kernel (blue) and stepped kernel (magenta); the prior distribution is shown in cyan. (TIF) [file pcbi.1005470.s009.tif]

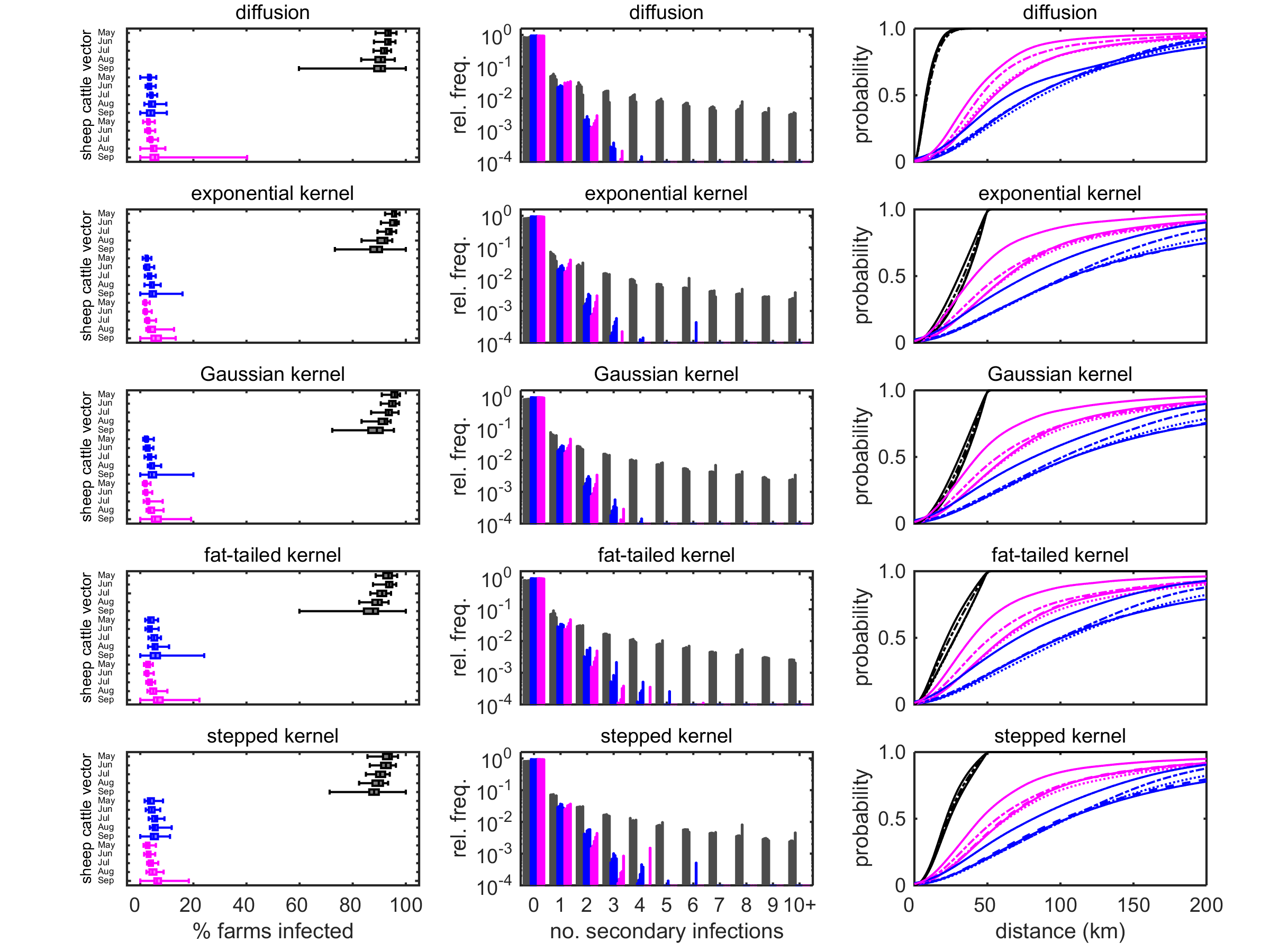

Supplement: S7 Fig — Left-hand column: proportion (%) of farms which are infected via dispersal of infected vectors (grey) or movement of infected cattle (blue) or sheep (magenta). Box-and-whisker plots show the posterior median (horizontal line), interquartile range (box) and 2.5th and 97.5th percentiles (whiskers). Middle column: frequency distribution for infected farms generating a number of secondary infections via vector dispersal (grey), movement of infected cattle (blue) or movement of infected sheep (magenta). Bars are for incursions in (from left to right) May, June, July, August or September. Right-hand column: cumulative density function for the distance between source and recipient farms when transmission occurs via vector dispersal (black), movement of infected cattle (blue) or movement of infected sheep (magenta). Lines are for incursions in May (right-most solid), June (dashed), July (dotted), August (dot-dashed) or September (left-most solid). Results are based on 100 replicates of the model identified in the panel title with parameters sampled from the joint posterior distribution. (TIF) [file pcbi.1005470.s010.tif]

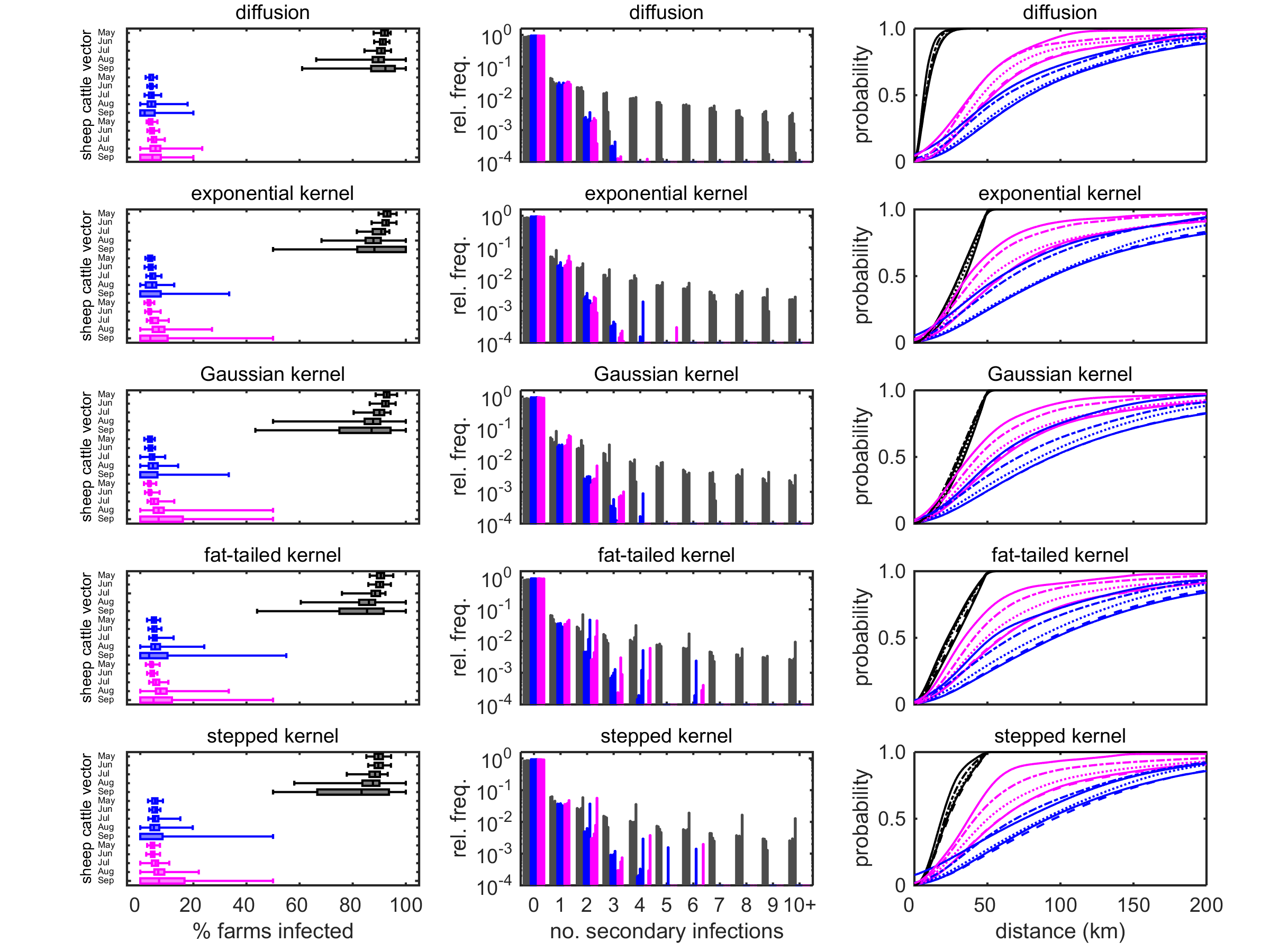

Supplement: S8 Fig — Panels are the same as in S7 Fig. (TIF) [file pcbi.1005470.s011.tif]

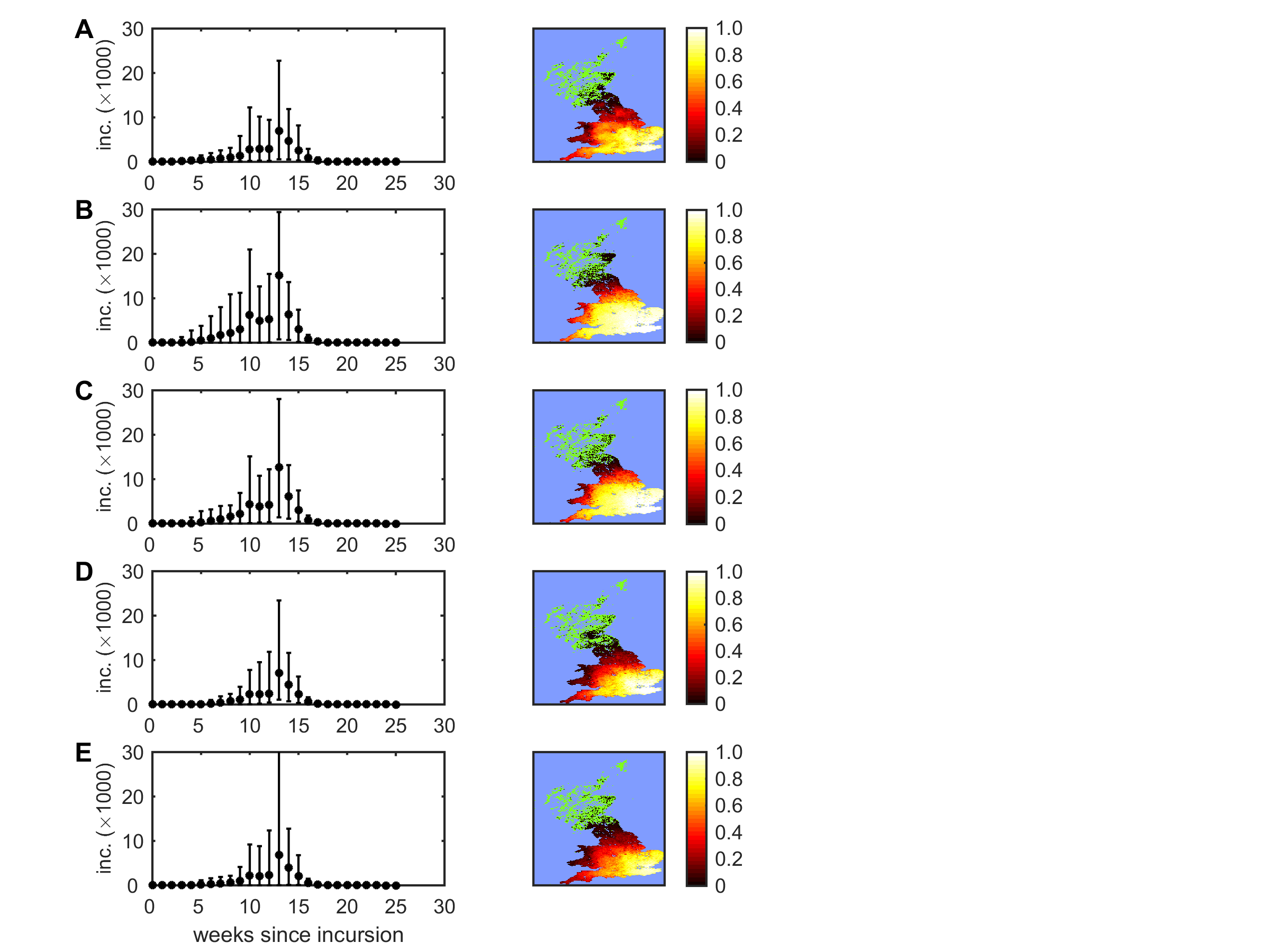

Supplement: S9 Fig — Simulated outbreaks are shown for the model in which vector dispersal is described by (A) a diffusion process, (B) an exponential kernel, (C) a Gaussian kernel, (D) a fat-tailed kernel or (E) a stepped kernel. The left-hand column shows the mean (circles) and 95% prediction intervals for the weekly incidence (number of newly infected farms). The right-hand column shows the predicted spatial spread of SBV. The map shows the cumulative probability of infection (see scale bar) expressed as the proportion of simulated outbreaks for which at least one farm was affected by SBV within each 5 km grid square. Results are based on 100 replicates of each model with parameters sampled from the joint posterior distribution. (TIF) [file pcbi.1005470.s012.tif]

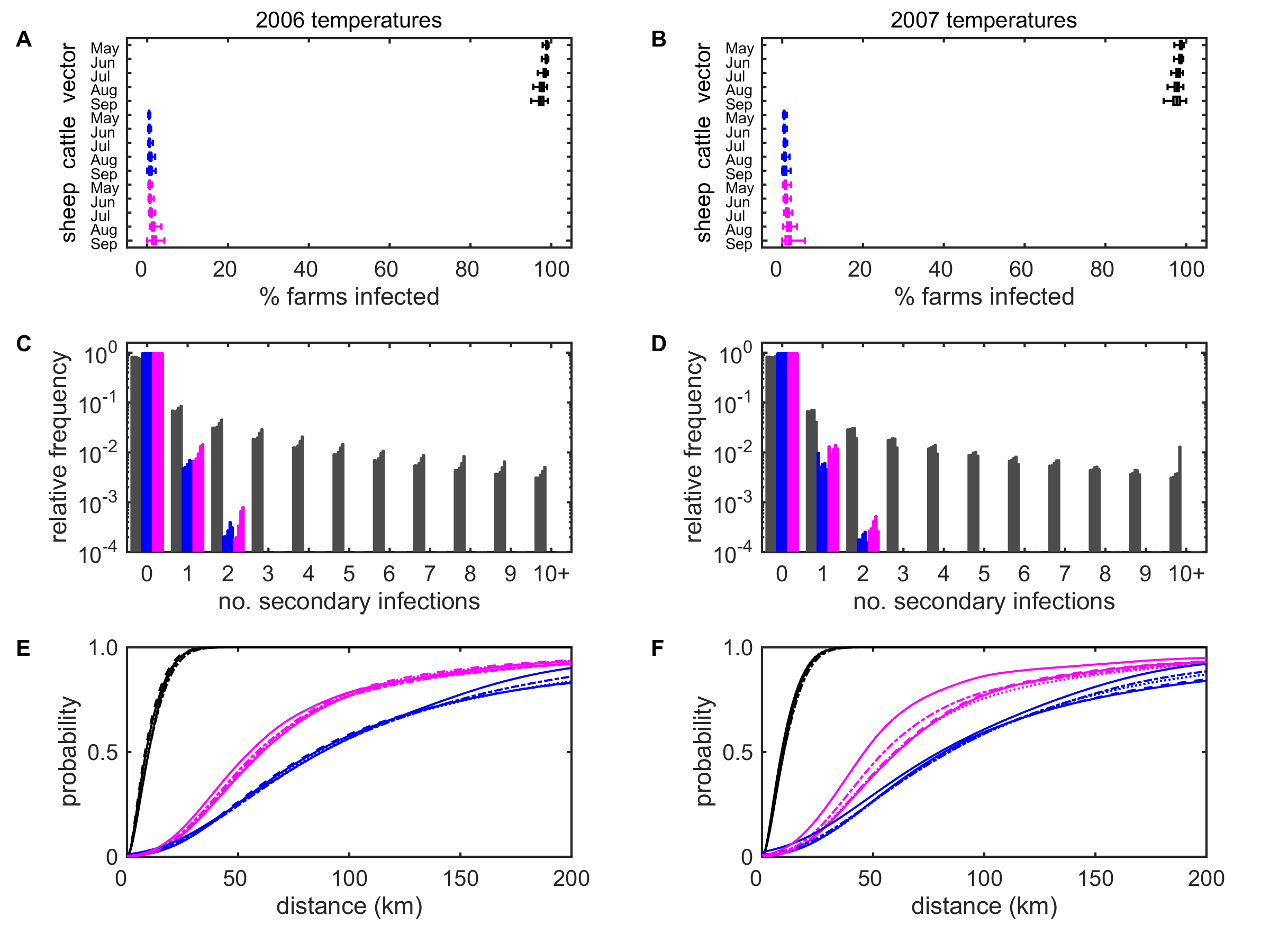

Supplement: S10 Fig — (A,B) Proportion (%) of farms which are infected via dispersal of infected vectors (grey) or movement of infected cattle (blue) or sheep (magenta). Box-and-whisker plots show the posterior median (horizontal line), interquartile range (box) and 2.5th and 97.5th percentiles (whiskers). (C,D) Frequency distribution for infected farms generating a number of secondary infections via vector dispersal (grey), movement of infected cattle (blue) or movement of infected sheep (magenta). Bars are for incursions in (from left to right) May, June, July, August or September. (E,F) Cumulative density function for the distance between source and recipient farms when transmission occurs via vector dispersal (black), movement of infected cattle (blue) or movement of infected sheep (magenta). Lines are for incursions in May (right-most solid), June (dashed), July (dotted), August (dot-dashed) or September (left-most solid). Results are based on 100 replicates of the diffusion model with parameters sampled from the joint posterior distribution using temperature data for (A,C,E) 2006 or (B,D,F) 2007. (TIF) [file pcbi.1005470.s013.tif]

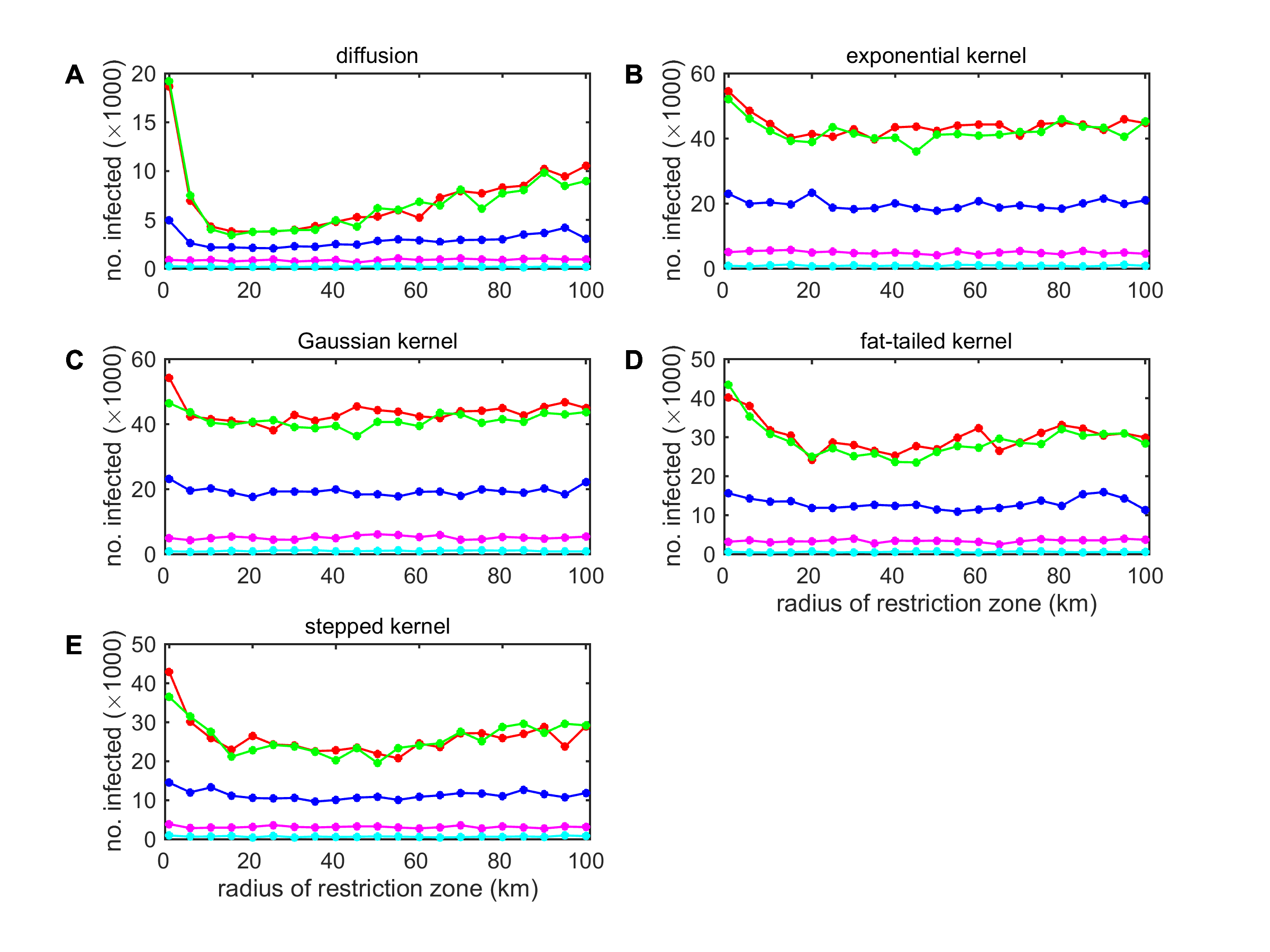

Supplement: S11 Fig — Each figure shows the mean cumulative number of infected farms (circles and lines) for simulations assuming different models for vector dispersal: (A) diffusion; (B) exponential kernel; (C) Gaussian kernel; (D) fat-tailed kernel; or (E) stepped kernel. Colour indicates the time of incursion: 1 May (red), 1 June (green), 1 July (blue), 1 August (magenta) or 1 September (cyan). For each scenario 100 replicates of the model were simulated using movement data for 2006 and temperature data for 2006. (TIF) [file pcbi.1005470.s014.tif]

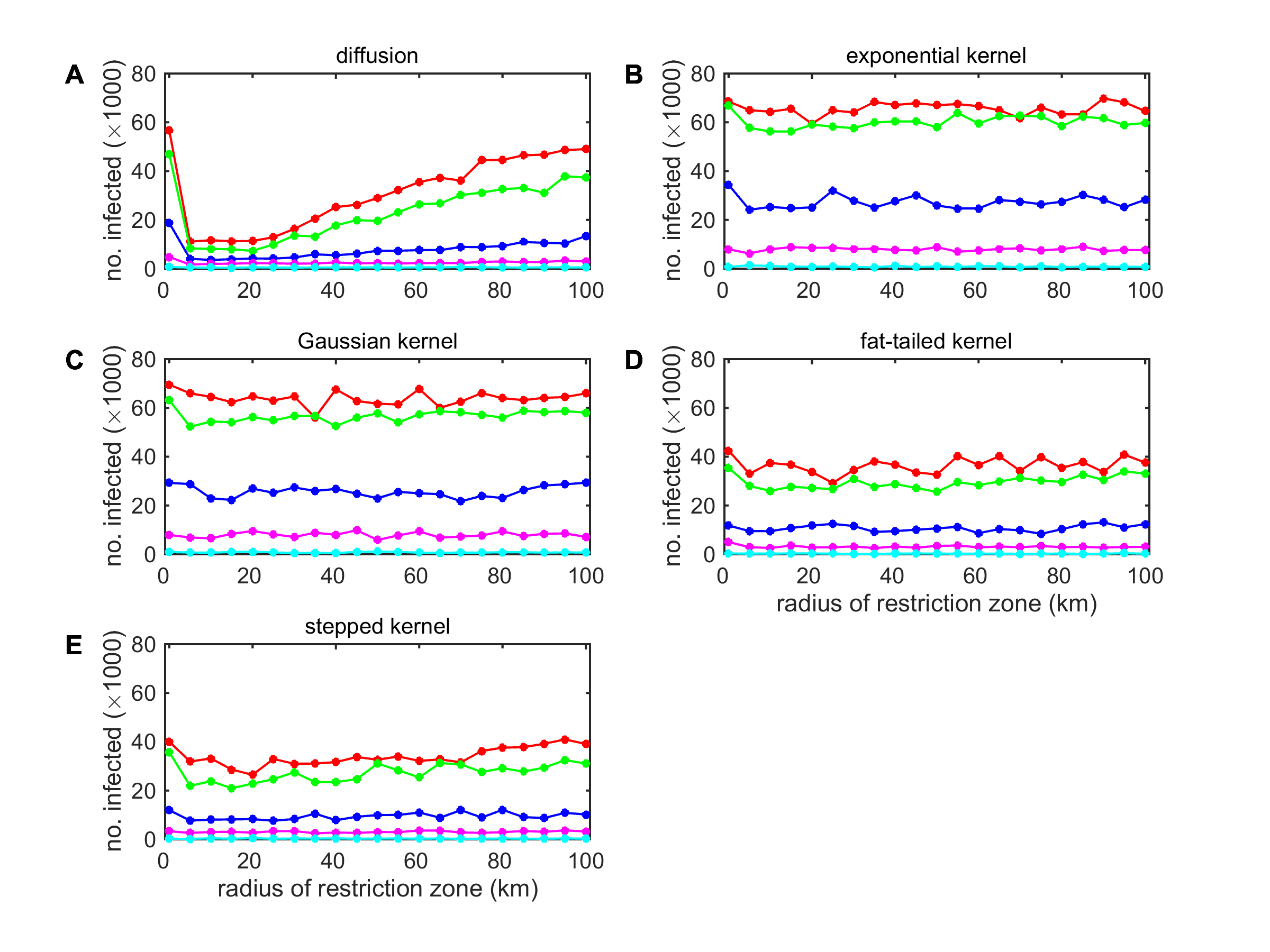

Supplement: S12 Fig — Each figure shows the mean cumulative number of infected farms (circles and lines) for simulations assuming different models for vector dispersal: (A) diffusion; (B) exponential kernel; (C) Gaussian kernel; (D) fat-tailed kernel; or (E) stepped kernel. Colour indicates the time of incursion: 1 May (red), 1 June (green), 1 July (blue), 1 August (magenta) or 1 September (cyan). For each scenario 100 replicates of the model were simulated using movement data for 2006 and temperature data for 2007. (TIF) [file pcbi.1005470.s015.tif]
