# Supplementary material for: Quantifying the roles of host movement and vector dispersal in the transmission of vector-borne diseases of livestock
Source: PLoS Comput Biol. 2017 Apr 3;13(4):e1005470. doi: 10.1371/journal.pcbi.1005470 (PMC5393902; doi:10.1371/journal.pcbi.1005470)
Supplement: S1 Table — (DOCX) [file pcbi.1005470.s016.docx]

**S1 Table.** Parameters in the model for the transmission of bluetongue virus within a farm.

| description | | | | symbol | prior or function* | comments and references |
| --- | --- | --- | --- | --- | --- | --- |
| probability of transmission from vector to host | | | | *b* | Beta(7.38,2.12) | distribution derived from analysis [1] of data on transmission of BTV to sheep by *C. sonorensis* [2] |
| probability of transmission from host to vector | | | | *β* | Beta(0.99,39.8) | distribution derived from data on experimental infection of field-caught *Culicoides* spp. in GB [3,4] using methods described in [1] |
| vectors to host ratio for species *i*  (cattle (*C*) or sheep (*S*)) | | | | *m_i_* | Gamma(*s_V_*,*µ_V_*/*s_V_*) | varies amongst farms |
| mean vector to host ratio | | | | *µ_V_* | Gamma(2,1000) | based on a maximum host biting rate (*m_i_a*) of 2500 bites per host per day [5]; cf. median holding size of 60 breeding cattle (census data) and light trap catches of up to 10000 midges per trap night [6] |
| shape parameter for vector to host ratio | | | | *s_V_* | Gamma(2,1) | - |
| number of animals of species *i* on farm | | | | *H_i_* | - | obtained from agricultural survey data |
| proportion of bites on species *i* | | | | *ϕ_i_* | - | for cattle *ϕ_C_*=*H_C_*/(*H_C_*+*σH_S_*), while for sheep *ϕ_S_*=*σH_S_*/(*H_C_*+*σH_S_*) |
| vector preference for sheep relative to cattle | | | | *σ* | Uniform(0,1) | vectors are assumed to feed preferentially on cattle [7,8] |
| reciprocal of the time interval between blood meals | | | | *a* | *a*(*T*)=0.0002*T*(*T*-3.7)(41.9-*T*)^1/2.7^ | depends on temperature [9] |
| duration of viraemia (cattle) | | | mean | 1/*r_C_* | N(20.6,1.6) | parameters for the priors estimated by fitting a gamma distribution to data on naturally infected cattle [10]; *n_C_* is constrained to take integer values |
|  |  |  | no. stages | *n_C_* | N(4.7,0.9) |  |
| disease-associated mortality rate (cattle) | | | | *d_C_* | Exponential(0.0025) | prior mean based on case fatality (5%) during the BT outbreak in the Netherlands in 2007 [11] |
| duration of viraemia (sheep) | | mean | | 1/*r_S_* | N(16.4,1.7) | parameters for the priors computed by fitting a gamma distribution to data on experimentally infected sheep [12,13] ; *n_S_* is constrained to take integer values |
|  |  | no. stages | | *n_S_* | Gamma(5,2.83) |  |
| disease-associated mortality rate (sheep) | | | | *d_S_* | Exponential(0.015) | prior mean based on case fatality (22%) during the BT outbreak in the Netherlands in 2007 [11] |
| extrinsic incubation period (EIP) | | | mean | 1/*ν* | *ν*(*T*)=*α*(*T*-*T*_min_) | reciprocal of mean EIP depends on temperature [14]; prior distributions derived from data on experimental infection of *C. sonorensis* with BTV-9 [14]; *k* is constrained to take integer values |
|  |  |  | no. stages | *k* | log N(2.65,0.77) |  |
| virus replication rate | | | | *α* | N(0.0190,0.0035) |  |
| threshold temperature for virus replication | | | | *T*_min_ | N(13.35,0.38) |  |
| vector mortality rate | | | | *μ* | *μ*(*T*)=0.009exp(0.16*T*) | depends on temperature [15] |
| vector recruitment rate | | | | ρ | - | for simplicity, assumed to be equal to equal to vector mortality rate |
| vector population size | | | | *N* | - | for simplicity, assumed to be constant; given by *N*=*m_i_H_i_* |
| vector activity | sin, 12 month | | | *b*_11_ | N(-1.56,0.17) | distributions derived from analysis of data collected from a network of 12 suction traps in England [16] |
|  | cos, 12 month | | | *b*_21_ | N(-3.74,0.50) |  |
|  | sin, 6 month | | | *b*_12_ | N(-1.49,0.11) |  |
|  | cos, 6 month | | | *b*_22_ | N(-1.00,0.35) |  |

* only those parameters with a specified prior distribution were estimated as part of the approximate Bayesian computation scheme

**References for S1 Table**

1. Lo Iacono G, Robin CA, Newton JR, Gubbins S, Wood JLN. Where are the horses? With the sheep or cows? Uncertain host location, vector feeding preferences and the risk of African horse sickness transmission in Great Britain. J R Soc Interface. 2013;10: 20130194.
2. Baylis M, O’Connell L, Mellor PS. Rates of bluetongue virus transmission between *Culicoides sonorensis* and sheep. Med Vet Entomol. 2008;22: 228-237.
3. Carpenter S, Lunt HL, Arav D, Venter GJ, Mellor PS. Oral susceptibility to bluetongue virus of *Culicoides* (Diptera: Ceratopogonidae) from the United Kingdom. J Med Entomol. 2006;43: 73-78.
4. Carpenter S, McArthur C, Selby R, Ward R, Nolan DV, Mordue Luntz AJ, Dallas JF, Tripet F, Mellor PS. Experimental infection studies of UK *Culicoides* species with bluetongue virus serotypes 8 and 9. Vet Record. 2008;163: 589-592.
5. Gerry AC, Mullens BA, MacLachlan NJ, Mecham OJ. Seasonal transmission of bluetongue virus by *Culicoides sonorensis* (Diptera: Ceratopogonidae) at a southern California dairy and evaluation of vectorial capacity as a predictor of bluetongue virus transmission. J Med Entomol. 2001;38: 197-209.
6. Meiswinkel R, Goffredo M, Leijs P, Conte A. The *Culicoides* ‘snapshot’: a novel approach used to assess vector densities widely and rapidly during the 2006 outbreak of bluetongue (BT) in The Netherlands. Prev Vet Med. 2008;87: 98-118.
7. Ayllón T, Nijhof AM, Weiher W, Bauer B, Allène X, Clausen P-H. Feeding behaviour of *Culicoides* spp. (Diptera: Ceratopogonidae) on cattle and sheep in northeast Germany. Parasites & Vectors. 2014;7: 34.
8. Elbers ARW, Meiswinkel R. *Culicoides* (Diptera: Ceratopogonidae) host preferences and biting rates in the Netherlands: Comparing cattle, sheep and black-light suction trap. Vet Parasitol. 2014;205: 330-337.
9. Mullens BA, Gerry AC, Lysyk TJ, Schmidtmann ET. Environmental effects on vector competence and virogenesis of bluetongue virus in *Culicoides*: interpreting laboratory data in a field context. Vet Ital. 2004;40: 160-166.
10. Melville LF, Weir R, Harmsen M, Walsh S, Hunt NT, Daniels PW. Characteristics of naturally occurring bluetongue viral infections of cattle. In St George TD, Kegao P, editors. Bluetongue disease in Southeast Asia and the Pacific. Canberra: ACIAR; 1996. pp. 245-250
11. Elbers ARW, van der Spek AN, van Rijn PA. Epidemiological characteristics of bluetongue virus serotype 8 laboratory confirmed outbreaks in The Netherlands in 2007 and a comparison with the situation in 2006. Prev Vet Med. 2009;92: 1-8.
12. Goldsmit L, Barzilai E, Tadmor A. 1975 The comparative sensitivity of sheep and chicken embryos to bluetongue virus and observations of viraemia in experimentally infected sheep. Aus Vet J. 1975;51: 190-196.
13. Veronesi E, Hamblin C, Mellor PS. Live attenuated bluetongue vaccine viruses in Dorset Poll sheep, before and after passage in vector midges (Diptera: Ceratopogonidae). Vaccine. 2005;23: 5509-5516.
14. Carpenter S, Wilson A, Barber J, Veronesi E, Mellor P, Venter G, Gubbins S. Temperature dependence of the extrinsic incubation period of orbiviruses in *Culicoides* biting midges. PLoS ONE. 2011;6: e27987.
15. Gerry AC, Mullens BA. Seasonal abundance and survivorship of *Culicoides sonorensis* (Diptera: Ceratopogonidae) at a southern Californian dairy, with reference to potential bluetongue virus transmission and persistence. J Med Entomol. 2000;37: 675-688.
16. Sanders CJ, Shortall C, Gubbins S, Burgin L, Gloster J, Harrington R, Reynolds DR, Mellor PS, Carpenter ST. Influence of season and meteorological parameters on flight activity of *Culicoides* biting midges in the United Kingdom. J Appl Ecol. 2011;48: 1355-1364.
