# Supplementary material for: Quantifying the roles of host movement and vector dispersal in the transmission of vector-borne diseases of livestock
Source: PLoS Comput Biol. 2017 Apr 3;13(4):e1005470. doi: 10.1371/journal.pcbi.1005470 (PMC5393902; doi:10.1371/journal.pcbi.1005470)
Supplement: S2 Table — (DOCX) [file pcbi.1005470.s017.docx]

**S2 Table.** Transitions, probabilities and population sizes in the model for the transmission of bluetongue virus within a farm.

| description | transition | probability | population size |
| --- | --- | --- | --- |
| *Hosts* |  |  |  |
| infection | $\left\{\begin{matrix} X^{(i)}\to X^{(i)}-1 \\ Y_{1}^{(i)}\to Y_{1}^{(i)}+1 \end{matrix}\right.$ | *λ_i_*δ*t* | __ |
| completion of infection stage *j*  (*j*=1,…,*n_i_*-1) | $\left\{\begin{matrix} Y_{j}^{(i)}\to Y_{j}^{(i)}-1 \\ Y_{j+1}^{(i)}\to Y_{j+1}^{(i)}+1 \end{matrix}\right.$ | *n_i_r_i_*δ*t* | __ |
| mortality during infection stage *j*  (*j*=1,…,*n_i_*) | $Y_{j}^{(i)}\to Y_{j}^{(i)}-1$ | *d_i_*δ*t* | __ |
| recovery | $\left\{\begin{matrix} Y_{n_{i}}^{(i)}\to Y_{n_{i}}^{(i)}-1 \\ Z^{(i)}\to Z^{(i)}+1 \end{matrix}\right.$ | *n_i_r_i_*δ*t* | __ |
| *Vectors* |  |  |  |
| infection | $\left\{\begin{matrix} S\to S-1 \\ L_{1}\to L_{1}+1 \end{matrix}\right.$ | *λ_V_*δ*t* | $S$ |
| completion of extrinsic incubation period (EIP), stage *j* (*j*=1,…,*k*-1) | $\left\{\begin{matrix} L_{j}\to L_{j}-1 \\ L_{j+1}\to L_{j+1}+1 \end{matrix}\right.$ | *kν*δ*t* | $L_{j}$ |
| vector mortality during EIP (*j*=1,…,*k*)  (and compensatory recruitment) | $\left\{\begin{matrix} L_{j}\to L_{j}-1 \\ S\to S+1 \end{matrix}\right.$ | *μ*δ*t* | $L_{j}$ |
| completion of EIP | $\left\{\begin{matrix} L_{k}\to L_{k}-1 \\ I\to I+1 \end{matrix}\right.$ | *k*νδ*t* | $L_{k}$ |
| mortality of infectious vectors  (and compensatory recruitment) | $\left\{\begin{matrix} I\to I-1 \\ S\to S+1 \end{matrix}\right.$ | *μ*δ*t* | $I$ |
