# Supplementary material for: Quantifying the roles of host movement and vector dispersal in the transmission of vector-borne diseases of livestock
Source: PLoS Comput Biol. 2017 Apr 3;13(4):e1005470. doi: 10.1371/journal.pcbi.1005470 (PMC5393902; doi:10.1371/journal.pcbi.1005470)
Supplement: S3 Table — (DOCX) [file pcbi.1005470.s018.docx]

**S3 Table.** Parameters in the logistic regression models for the probability of a farm moving (off move) or receiving (on move) cattle and sheep.

| parameter | moving | | receiving | |
| --- | --- | --- | --- | --- |
|  | cattle | sheep | cattle | sheep |
| intercept | -4.77 | -4.71 | -4.95 | -6.33 |
| no. animals | 1.96×10^-3^ | 4.48×10^-4^ | 2.13×10^-3^ | 3.38×10^-4^ |
| month |  |  |  |  |
| January | 0 | 0 | 0 | 0 |
| February | 0.15 | -0.15 | 0.20 | -8.21×10^-3^ |
| March | 0.27 | -0.19 | 0.31 | 0.17 |
| April | 0.46 | -0.13 | 0.54 | 0.11 |
| May | 0.46 | 4.44×10^-3^ | 0.58 | 9.42×10^-2^ |
| June | 0.25 | 4.96×10^-3^ | 0.36 | -0.15 |
| July | 0.13 | 6.30×10^-2^ | 0.21 | 0.18 |
| August | 0.19 | 0.48 | 0.22 | 0.11 |
| September | 0.29 | 0.77 | 0.35 | 0.19 |
| October | 0.42 | 0.70 | 0.53 | 0.16 |
| November | 0.48 | 0.51 | 0.50 | 0.78 |
| December | 1.31×10^-2^ | 4.52×10^-2^ | -1.99×10^-3^ | 0.11 |
