# Supplementary material for: Quantifying the roles of host movement and vector dispersal in the transmission of vector-borne diseases of livestock
Source: PLoS Comput Biol. 2017 Apr 3;13(4):e1005470. doi: 10.1371/journal.pcbi.1005470 (PMC5393902; doi:10.1371/journal.pcbi.1005470)
Supplement: S4 Table — (DOCX) [file pcbi.1005470.s019.docx]

**S4 Table.** Probability distribution for the number of batches of cattle or sheep moved off a farm which moves any livestock on a given day.

| no. batches | 1 | 2 | 3 | 4 | 5 | 6 | 7 |
| --- | --- | --- | --- | --- | --- | --- | --- |
| cattle | 0.62 | 0.18 | 0.09 | 0.05 | 0.03 | 0.02 | 0.01 |
| sheep | 0.97 | 0.03 | 0 | 0 | 0 | 0 | 0 |
