# Supplementary material for: Quantifying the roles of host movement and vector dispersal in the transmission of vector-borne diseases of livestock
Source: PLoS Comput Biol. 2017 Apr 3;13(4):e1005470. doi: 10.1371/journal.pcbi.1005470 (PMC5393902; doi:10.1371/journal.pcbi.1005470)
Supplement: S5 Table — (DOCX) [file pcbi.1005470.s020.docx]

**S5 Table.** Posterior mean and 95% credible intervals for parameters in the diffusion and kernel models for the transmission of bluetongue virus between farms.

| description | symbol | mean | 95% credible interval | |
| --- | --- | --- | --- | --- |
|  |  |  | lower | upper |
| *diffusion model* |  |  |  |  |
| transmission parameter | *γ* | 0.62 | 0.23 | 0.94 |
| diffusion rate | *D* | 3.01 | 1.04 | 4.67 |
| *exponential kernel* |  |  |  |  |
| transmission parameter | *γ* | 1.63 | 0.69 | 2.78 |
| kernel parameter | *α* | 1.4×10^-2^ | 1.2×10^-3^ | 3.3×10^-2^ |
| *Gaussian kernel* |  |  |  |  |
| transmission parameter | *γ* | 1.45 | 0.62 | 2.42 |
| kernel parameter | *α* | 3.0×10^-4^ | 2.0×10^-5^ | 7.6×10^-4^ |
| *fat-tailed kernel* |  |  |  |  |
| transmission parameter | *γ* | 1.83 | 0.90 | 2.91 |
| kernel power | *α* | 1.73 | 1.06 | 3.03 |
| distance scaling | *d*_0_ | 13.35 | 5.80 | 21.83 |
| *stepped kernel* |  |  |  |  |
| transmission parameter | *γ* | 1.39 | 0.63 | 2.35 |
| kernel power | *α* | 2.97 | 1.22 | 4.74 |
| distance parameter | *d*_0_ | 18.70 | 9.76 | 28.30 |
