# Supplementary material for: Quantifying the roles of host movement and vector dispersal in the transmission of vector-borne diseases of livestock
Source: PLoS Comput Biol. 2017 Apr 3;13(4):e1005470. doi: 10.1371/journal.pcbi.1005470 (PMC5393902; doi:10.1371/journal.pcbi.1005470)
Supplement: S6 Table — (DOCX) [file pcbi.1005470.s021.docx]

**S6 Table.** Posterior mean and 95% credible intervals for parameters in the model† for the transmission of bluetongue virus within farms.

| description | | symbol | mean | 95% credible interval | |
| --- | --- | --- | --- | --- | --- |
|  |  |  |  | lower | upper |
| *under-ascertainment* | |  |  |  |  |
| daily probability of reporting (cattle) | | *ζ_C_* | 9.5×10^-3^ | 5.2×10^-4^ | 2.6×10^-2^ |
| daily probability of reporting (sheep) | | *ζ_S_* | 1.3×10^-2^ | 7.3×10^-4^ | 3.5×10^-2^ |
| *within-farm transmission* | |  |  |  |  |
| probability of transmission from vector to host | | *b* | 0.84 | 0.68 | 0.96 |
| probability of transmission from host to vector | | *β* | 0.023 | 0.0073 | 0.042 |
| mean vector-to-host ratio | | *μ_V_* | 1806.7 | 688.2 | 3141.1 |
| shape parameter for vector-to-host ratio | | *s_V_* | 1.69 | 0.54 | 3.17 |
| vector preference for sheep relative to cattle | | *σ* | 0.095 | 0.0029 | 0.40 |
| mean duration of viraemia (cattle) | | 1/*r*_C_ | 20.53 | 18.76 | 22.26 |
| shape parameter for duration of viraemia (cattle) | | *n_C_* | 5.3 | 4 | 6 |
| disease-associated mortality rate (cattle) | | *d_C_* | 1.2×10^-3^ | 1.2×10^-4^ | 2.9×10^-4^ |
| mean duration of viraemia (sheep) | | 1/*r_S_* | 16.10 | 14.12 | 18.20 |
| shape parameter for duration of viraemia (sheep) | | *n_S_* | 12.4 | 6 | 20 |
| disease-associated mortality rate (sheep) | | *d_S_* | 6.8×10^-3^ | 5.9×10^-4^ | 1.7×10^-2^ |
| virus replication rate | | *α* | 0.020 | 0.016 | 0.024 |
| threshold temperature for virus replication | | *T*_min_ | 13.2 | 12.8 | 13.7 |
| no. stages in extrinsic incubation period (EIP) | | *k* | 11 | 3 | 22 |
| vector activity | sin, 12 month | *b*_11_ | -1.59 | -1.80 | -1.38 |
|  | cos, 12 month | *b*_21_ | -3.80 | -4.37 | -3.22 |
|  | sin, 6 month | *b*_12_ | -1.46 | -1.60 | -1.32 |
|  | cos, 6 month | *b*_22_ | -0.99 | -1.38 | -0.60 |

† These estimates are for the model in which vector dispersal is described using a diffusion process.
